# Genome-Wide Linkage and Association Study of Childhood Gender Nonconformity in Males

**DOI:** 10.1007/s10508-021-02146-x

**Published:** 2021-09-13

**Authors:** Alan R. Sanders, Gary W. Beecham, Shengru Guo, Khytam Dawood, Gerulf Rieger, Ritesha S. Krishnappa, Alana B. Kolundzija, J. Michael Bailey, Eden R. Martin

**Affiliations:** 1 Department of Psychiatry and Behavioral Sciences, NorthShore University HealthSystem Research Institute, 1001 University Place, Evanston, IL 60201 USA; 2 Department of Psychiatry and Behavioral Neuroscience, University of Chicago, Chicago, IL USA; 3 John P. Hussman Institute for Human Genomics, University of Miami Miller School of Medicine, Miami, FL USA; 4 Dr. John T. Macdonald Foundation Department of Human Genetics, University of Miami Miller School of Medicine, Miami, FL USA; 5 Department of Psychology, Pennsylvania State University, University Park, PA USA; 6 Department of Psychology, University of Essex, Colchester, UK; 7 Department of Psychiatry, Icahn School of Medicine at Mount Sinai, Elmhurst, NY USA; 8 Collective Impact, Washington, DC USA; 9 Department of Psychology, Northwestern University, Evanston, IL USA

**Keywords:** Complex trait, Genome-wide linkage scan, Male sexual orientation, Childhood gender nonconformity, GWAS

## Abstract

Male sexual orientation is influenced by environmental and complex genetic factors. Childhood gender nonconformity (CGN) is one of the strongest correlates of homosexuality with substantial familiality. We studied brothers in families with two or more homosexual brothers (409 concordant sibling pairs in 384 families, as well as their heterosexual brothers), who self-recalled their CGN. To map loci for CGN, we conducted a genome-wide linkage scan (GWLS) using SNP genotypes. The strongest linkage peaks, each with significant or suggestive two-point LOD scores and multipoint LOD score support, were on chromosomes 5q31 (maximum two-point LOD = 4.45), 6q12 (maximum two-point LOD = 3.64), 7q33 (maximum two-point LOD = 3.09), and 8q24 (maximum two-point LOD = 3.67), with the latter not overlapping with previously reported strongest linkage region for male sexual orientation on pericentromeric chromosome 8. Family-based association analyses were used to identify associated variants in the linkage regions, with a cluster of SNPs (minimum association *p* = 1.3 × 10^–8^) found at the 5q31 linkage peak. Genome-wide, clusters of multiple SNPs in the 10^–6^ to 10^–8^ *p*-value range were found at chromosomes 5p13, 5q31, 7q32, 8p22, and 10q23, highlighting glutamate-related genes. This is the first reported GWLS and genome-wide association study on CGN. Further increasing genetic knowledge about CGN and its relationships to male sexual orientation should help advance our understanding of the biology of these associated traits.

**Supplementary Information:**

The online version contains supplementary material available at 10.1007/s10508-021-02146-x.

## Introduction

Male sexual orientation is moderately heritable (30 ~ 40% heritability) and appears multifactorial, with evidence of multiple genetic and environmental contributions via family, twin, and segregation analyses (Alanko et al., 2010; Bailey & Bell, 1993; Bailey & Benishay, 1993; Bailey & Pillard, 1991; Bailey et al., 1993, 1999, 2000; Buhrich et al., 1991; Hamer et al., 1993; Heston & Shields, 1968; Kallmann, 1952; Kendler et al., 2000; King & McDonald, 1992; Kirk et al., 2000; Langström et al., 2010; Pattatucci & Hamer, 1995; Pillard & Weinrich, 1986; Santtila et al., 2008; Schwartz et al., 2010; Whitam et al., 1993). Genome-wide linkage studies (GWLS) of homosexual brother pairs have been applied to the trait (Mustanski et al., 2005; Ramagopalan et al., 2010; Sanders et al., 2015), with the largest GWLS sample finding genome-wide significant linkage to the pericentromeric region of chromosome 8 (LOD = 4.08) and strong support for the previously reported linkage to Xq28 (LOD = 2.99) (Sanders et al., 2015). In addition, genome-wide association studies (GWAS) of the trait are now emerging (Drabant et al., 2012; Sanders et al., 2017); most recently, a GWAS with a greatly enlarged sample size found five loci (two in males, one in females, and two in the combined analyses) significantly associated with same-sex sexual behavior (Ganna et al., 2019).

Sexual orientation is empirically closely linked to some aspects of gender roles, including childhood play behavior and gender identity (Bailey & Zucker, 1995), and adult sex-typed behavior, particularly occupational and recreational interests (Lippa, 2008). While many studies (see below) have been retrospective, i.e., querying recalled childhood gender nonconformity (CGN), recent large-scale, population-based prospective studies have replicated the robust relationship between sexual orientation and CGN (Li et al., 2017; Xu et al., 2019, 2020, 2021). One complementary approach to prospective studies relies on analyzing behaviors depicted in home videos made during childhood, later blindly rated by rater panels; consistent with typical consolidation of gender identity, sexual orientation differences in observer rated CGN diverged around age 3 or 4 years (Rieger et al., 2008). A second approach has examined the correspondence between homosexual men’s recalled childhood gender nonconformity and their mothers’ memories of their sons, finding a strong association, *r* = 0.69 (Bailey et al., 1995). The strong association between sexual orientation and CGN is cross-culturally robust as well (Bailey et al., 2016). One meta-analysis of 41 retrospective studies showed that homosexuals recalled substantially more CGN than did heterosexuals (Bailey & Zucker, 1995). The effect sizes reported for both sexes are among the largest ever reported in the realm of sex-dimorphic behaviors—the Cohen’s *d* (Cohen, 1988) was 1.3 for men and 1.0 for women (Bailey & Zucker, 1995). In our family linkage sample (Sanders et al., 2015), we found moderate familiality and replicated CGN differences by sexual orientation in males (Bailey et al., 2020). Of further note, in twin studies, CGN shows moderate to high heritability in males: 37% (Knafo et al., 2005), 70% (van Beijsterveldt et al., 2006), 29% (Alanko et al., 2010), and 50% (Bailey et al., 2000). Despite these findings, there have not yet been gene mapping efforts for CGN. Here, we report the first GWLS and GWAS on CGN in males.

## Method

### Participants and Measures

We studied a set of families each with two or more homosexual brothers (409 concordant sibling pairs in 384 families) collected largely from community festivals for a linkage study on male sexual orientation and detailed previously (Sanders et al., 2015). The sample was predominantly of European ancestry (98%) and non-Hispanic (95%), and the average age of the brothers was 44 years old. Briefly, after obtaining written informed consent as approved by the institutional review board of NorthShore University HealthSystem, blood was collected for DNA and questionnaires were completed, including both on sexual orientation (Kinsey scales; (Kinsey et al., 1948) and CGN (Rieger et al., 2008; Zucker et al., 2006). For sexual orientation in the studied sample, homosexual men identified as homosexual and endorsed a Kinsey score of 5 or 6 for fantasy, while heterosexual men identified as heterosexual and endorsed a Kinsey score of 0 or 1 for fantasy. For CGN, we used a scale with 23 items: thirteen items from The Recalled Childhood Gender Identity/Gender Role Questionnaire (items 1, 2, 3, 5, 7, 8, 10, 11, 15, 18, 19, 20, and 21) (Zucker et al., 2006); all seven items from the Childhood Gender Nonconformity Scale (Men) (Rieger et al., 2008), and three items created for this study. The three new items were: (1) “Looking back, I think that others must have found me” (answers ranging from 1 = very masculine to 5 = very feminine); (2) “When I was a child, peers” (answers ranging from 1 = frequently commented on my masculinity to 5 = frequently commented on my femininity); and (3) “When I was a child, adults” (answers ranging from 1 = frequently commented on my masculinity to 5 = frequently commented on my femininity). All items were rescaled so that higher scores indicated greater CGN. Finally, items were standardized and then averaged, with higher scores indicating higher CGN and achieving a reliability (coefficient alpha) of 0.91 as detailed in our recent family study (Bailey et al., 2020). We had CGN scores for 824 of the 826 genotyped brothers. Since the CGN scores were not normally distributed (Shapiro–Wilk test; (Shapiro & Wilk, 1965), *p* < 0.0001), prior to genetic analyses below, we transformed CGN scores via sqrt(2 + CGN) to normalize them (i.e., normCGN, which yielded a Shapiro–Wilk test result of *p* = 0.61).

### Analyses

Genotyping (Affymetrix 5.0 SNP array) and rigorous quality control (QC) steps were previously detailed (Sanders et al., 2015, 2017). Briefly, these included (1) removal of SNPs (minor allele frequency, MAF < 0.05; missingness ≥ 1%; Hardy–Weinberg equilibrium [HWE] deviation *p* < 10^–6^), and (2) removal of samples (missingness > 5%; failing checks for duplications and relatedness; ancestry outliers via principal component analysis, PCA). Following the QC filter application in the larger dataset (Sanders et al., 2015, 2017), the families with individuals with CGN scores were extracted for analysis. We conducted our two-point and multipoint GWLS with MERLIN (Abecasis et al., 2002) using the variance components linkage analysis approach with CGN scores as a quantitative trait. For multipoint analysis, we used a set of 45,451 (44,778 autosomal, 673 chromosome X) SNPs that had been pruned for linkage disequilibrium (LD, r^2^ > 0.16) using PLINK v1.9 (Purcell et al., 2007) and MAF < 0.1. Prior to performing GWAS analyses, we imputed using the IMPUTE2 software (Howie et al., 2009) with the 1,000 Genomes Project Phase 3 reference data(Abecasis et al., 2010) (removing SNPs with an information score < 0.6, MAF < 0.05). We employed the R package, Genome-Wide Association analyses with Family (GWAF) (Chen & Yang, 2010) using the first three principal components (PCs) as covariates to conduct our GWAS of CGN on a final QC’d SNP dataset containing a total of 6,240,683 retained SNPs (210,033 typed and 6,030,650 imputed).

## Results

**Fig. 1:**
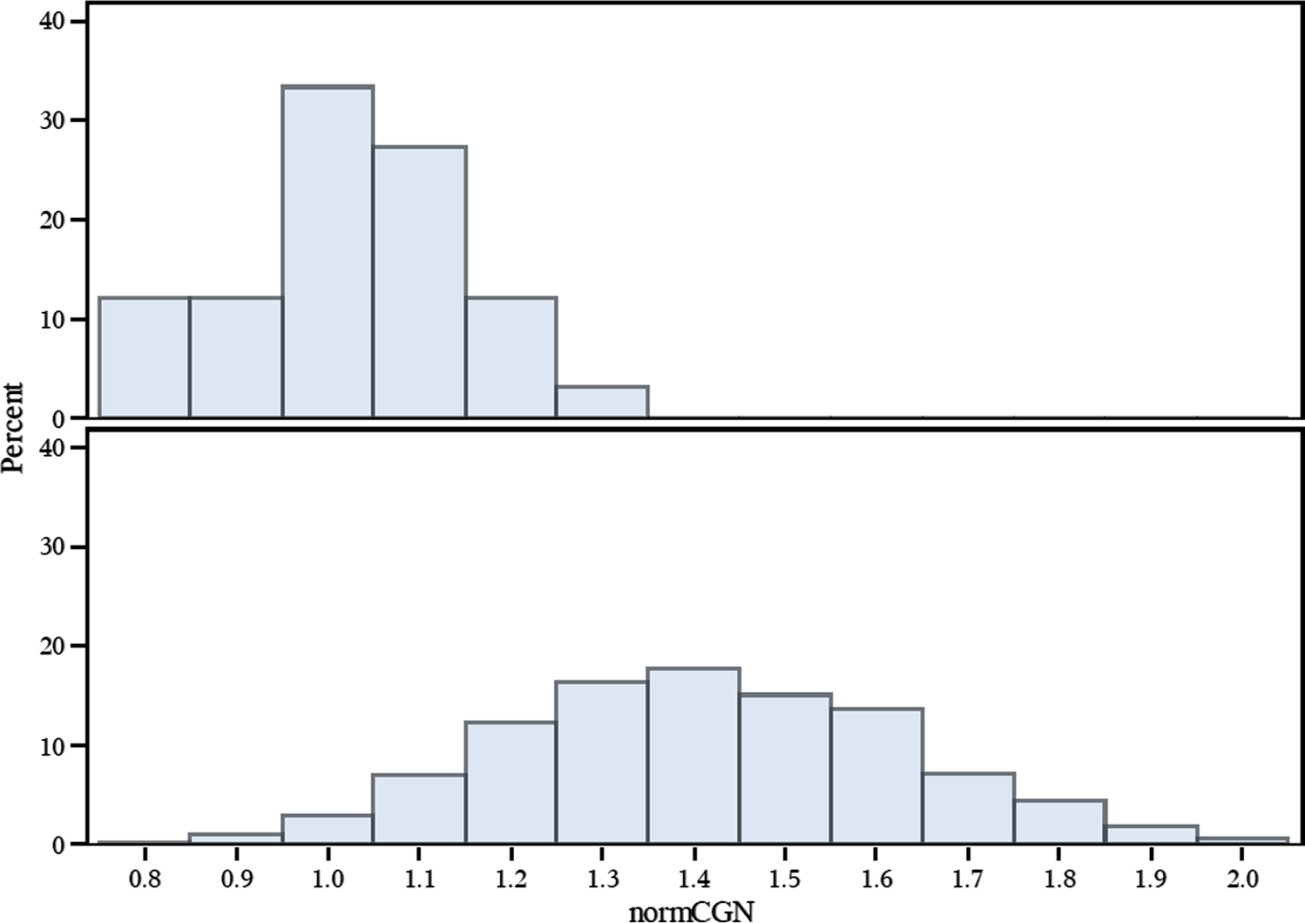
NormCGN distributions plotted by sexual orientation. normCGN score bins (x-axis) are plotted against percentage of men (y-axis) in two panels. The top panel of 33 heterosexual men and the bottom panel of 791 homosexual men

**Fig. 2:**
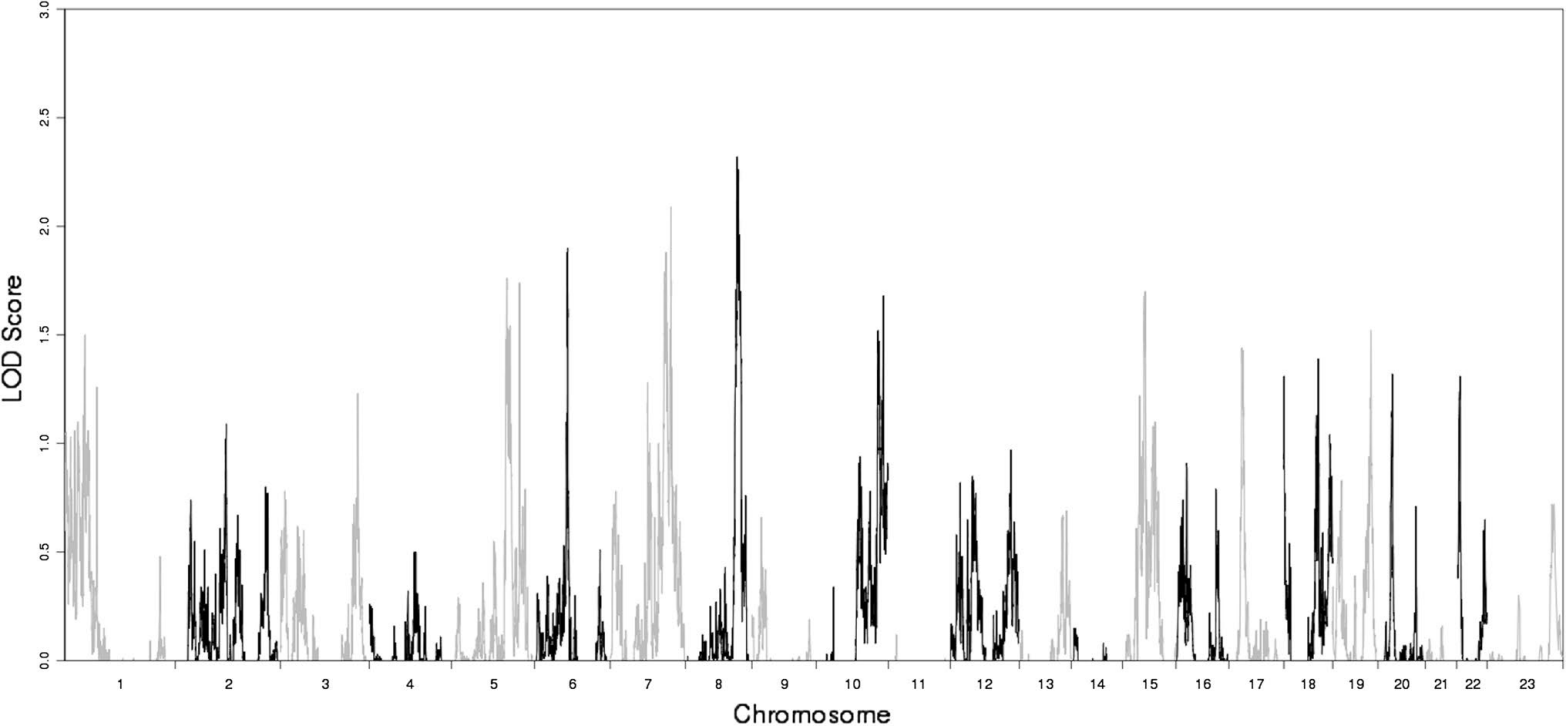
Multipoint LOD scores plotted by chromosomal positions for the GWLS of normCGN. Adjacent chromosomes are separated by alternating black and gray lines. Maximum multipoint LOD scores are at ∼129 cM on chromosome 8q24

The normCGN score distributions are plotted by sexual orientation (Fig. 1), with a recapitulation of the patterns previously seen (Bailey & Zucker, 1995), namely that scores were more variable for homosexual men (M = 1.41, variance = 0.046) and clustered more tightly in the gender conforming region of the distribution for heterosexual men (M = 1.02, variance = 0.017) with the intergroup differences being significant (*p* < 0.0001, *t*-test). In the GWLS, we detected suggestive two-point linkage (LOD ≥ 2.2) (Lander & Kruglyak, 1995) for 382 SNPs (Supplementary Table 1) with ten SNPs exceeding the threshold for genome-wide significance (LOD ≥ 3.6) (Lander & Kruglyak, 1995). Of these ten SNPs, a cluster of five SNPs (rs2349010, rs11242393, rs2074349, rs13172798, rs17171566) at chromosome 5q31 were intronic in *KLHL3* (*kelch like family member 3*), one (rs7841264) at 8q24 was intronic in *CASC8* (*cancer susceptibility 8*), and the other four (rs555920, rs877688, rs4416661, rs2967925) were intergenic. However, we note that linkage signals are imprecise (especially for traits manifesting complex genetics) and thus, larger regions containing additional genes are implicated. Our multipoint nonparametric GWLS results (Fig. 2) show the four strongest peaks (multipoint LOD > 1.8) located on chromosomes 5 (136 ~ 152 cM, based on multipoint drop-1 LOD support interval), 6 (79 ~ 86 cM),7 (139 ~ 158 cM), and 8 (127 ~ 138 cM); three of these peaks (chromosomes 5, 6, and 8) contain SNPs with genome-wide significant two-point LOD scores, with respective two-point LOD maxima being 4.45 (rs17171566), 3.64 (rs4416661), and 3.67 (rs7841264). We note that these locations do not overlap with previously reported linkage peaks for male sexual orientation, such as at pericentromeric chromosome 8 (Sanders et al., 2015).

**Fig. 3:**
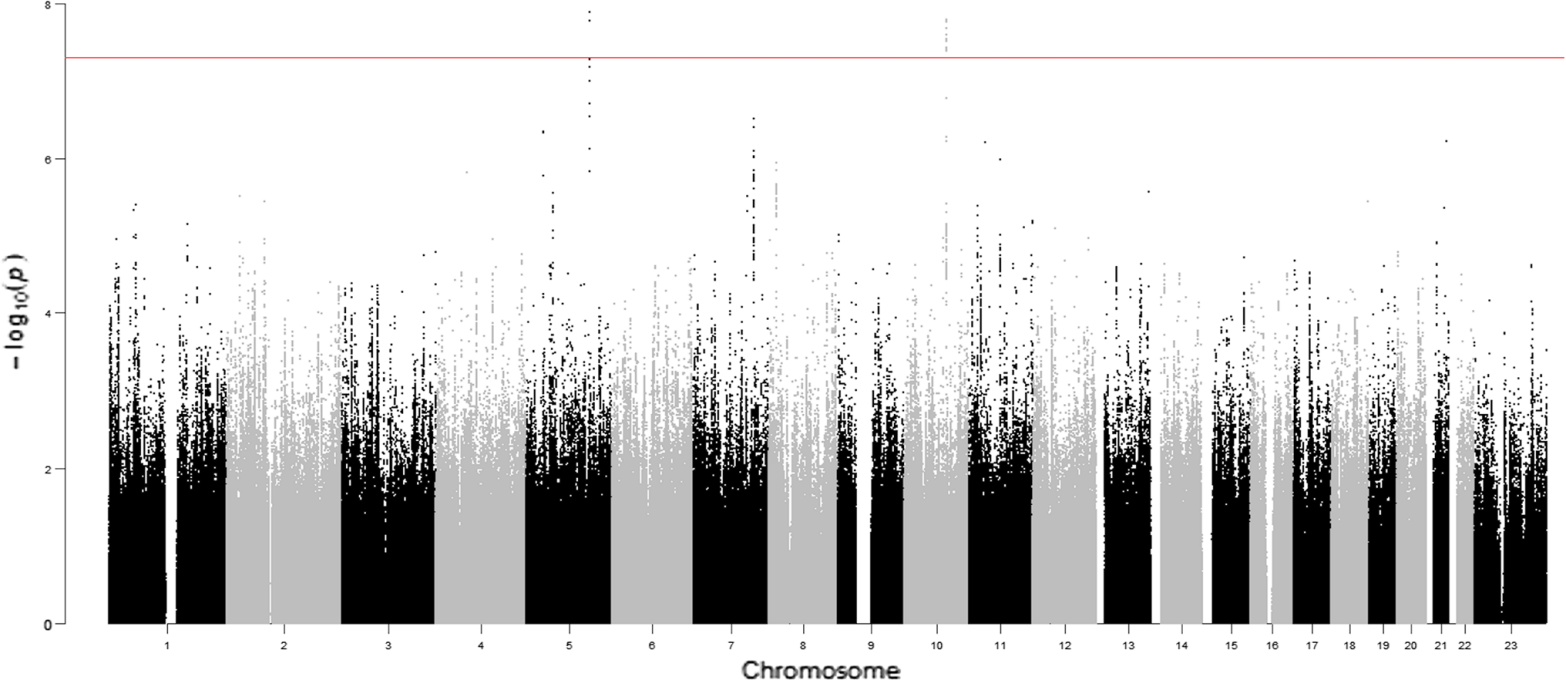
Manhattan plot of GWAS for normCGN. Plot of negative log_10_ of the *p*-values for the single SNP association analysis, ordered along the x-axis by chromosomal position

Our GWAS (Fig. 3) showed several regions of multiple SNPs in the 10^–6^ to 10^–8^ *p*-value range, including two regions with SNPs reaching genome-wide significance (5 × 10^–8^). For our GWAS QC, we achieved a λ_1000_ = 1.046 (i.e., a low genomic inflation factor; Supplementary Fig. 1 for quantile–quantile plot). The top regions (Supplementary Table 1) were on chromosomes 5p13 (minimum *p* = 4.4 × 10^–7^, rs3832338), 5q31 (minimum *p* = 1.3 × 10^–8^, rs113946051), 7q32 (minimum *p* = 3.1 × 10^–7^, rs3757757), 8p22 (minimum *p* = 1.2 × 10^–6^, rs17488730), and 10q23 (minimum *p* = 1.6 × 10^–8^, rs1008912). There are a number of genes of potential relevance to CGN in and around these regions, as described below. Regional association plots for the top linkage regions are displayed in Supplementary Figs. 2 (chromosome 5), 3 (chromosome 6), 4 (chromosome 7), and 5 (chromosome 8). Of note, the linkage peak on chromosome 5q31 also contains a cluster of associated (10^–6^ < *p* < 10^–8^ p-value) SNPs (Supplementary Table 2, Supplementary Fig. 2). In addition, we display a regional association plot for chromosome 10q23 (Supplementary Fig. 6), which though not in a top linkage region did show genome-wide significant association for 9 SNPs.

## Discussion

In this first GWLS on CGN in males, we found genome-wide significant linkage with multipoint support for several linkage regions, most notably at chromosomes 5q31 and 8q24 (Fig. 2, Supplementary Table 1). The strongest multipoint linkage peaks for CGN (Fig. 2) did not align with the strongest such signals from earlier GWLS on male sexual orientation (Sanders et al., 2015). This was not unexpected since while CGN and sexual orientation are associated phenotypes, they are far from being the same and both are traits with complex genetics, and thus, one would not necessarily expect largely overlapping linkage or association patterns. We note that one of the top multipoint peaks from the GWLS (chromosome 5q31, Supplementary Fig. 2) also contains a cluster of 10 associated (10^–6^ < *p* < 10^–8^ *p* value) SNPs from the GWAS, 2 of which are genome-wide significant associations, thus with both linkage and association positional evidence. However, none of the genes in the immediate region of this cluster have obvious putative connections to CGN.

This initial GWAS report on CGN had some interesting findings as well. Compared to the previous GWAS on male sexual orientation on the same dataset (Sanders et al., 2017), the current CGN GWAS had substantially more regions with SNPs associated at a level of 10^–6^ < *p* < 10^–8^, including two loci (5q31, 10q23) breaching genome-wide significance (Fig. 3, Supplementary Table 2). Possible explanations include a potentially stronger genetic contribution for CGN (versus sexual orientation) and enhanced statistical power for a quantitative measure with CGN (versus a categorical approach for sexual orientation). A recent large association meta-analysis of same-sex sexual behavior found five genome-wide significant loci (Ganna et al., 2019); however, none of those loci overlap with the top GWLS or GWAS findings for CGN in the current study.

We found two loci (5q31, 10q23) with SNPs reaching genome-wide significance (*p* < 5 × 10^–8^) for association with CGN and detected several additional regions (Fig. 3, Supplementary Table 2) with promising findings (10^–6^ < *p* < 10^–8^ association p-values). These regions contain a number of genes of putative relevance to the trait, some of which we highlight next. At the 5p13 SNP cluster, the nearest gene is *SLC1A3*, a brain expressed glutamate transporter which has been implicated in some behavioral phenotypes, e.g., attention deficit hyperactivity disorder, mood disorders, cortico-limbic connectivity during affective regulation (Huang et al., 2019; Medina et al., 2016; Poletti et al., 2018; van Amen-Hellebrekers et al., 2016). The 10q23 SNP cluster overlaps with *GRID1*, which encodes a glutamate receptor channel subunit, and has also been implicated in various behavioral phenotypes (e.g., mood disorders; Fallin et al., 2005; Zhang et al., 2018) and when deleted in the mouse leads to changes in emotional and social behaviors (Yadav et al., 2012). The SNPs in the 7q32 cluster fall within (3’UTR, synonymous coding) and near *LRRC4*, which has been implicated in autism spectrum disorders (Du et al., 2020; Um et al., 2018). When deleted (*Lrrc4*^−/−^) in the mouse, N-Methyl-D-aspartate receptor (NMDAR, an ionotropic glutamate receptor)-dependent synaptic plasticity in the hippocampus was decreased, and these mice displayed mild social interaction deficits, increased self-grooming, and modest anxiety-like behaviors, which were reversed by pharmacological NMDAR activation (Um et al., 2018). Thus, three of the top associated SNP clusters involve glutamate-related genes which have separate evidence of relevance to other behavioral traits, some of which vary in prevalence by gender (e.g., mood disorder; (Sanders et al., 2010) and references therein) in the general population.

Gene mapping challenges include those inherent to GWLS and GWAS of traits manifesting complex genetics such as CGN, as well as limitations in statistical power given the sample size. We discuss power limitations further in the supplementary text but note here that for traits manifesting complex genetics (such as CGN), contributory genetic variants generally have individually small effects, leading to challenges in generating replicable findings. Other limitations include the current study being on a predominantly European ancestry sample and only on males, using retrospective recall of CGN rather than prospective ratings, and not including a replication sample. Replication and extension efforts are somewhat hampered in that relevant survey questions are often not included in large biobank samples such as for CGN; however, there are more sexuality data-points becoming available in some instances (e.g., sexual orientation and gender identity questions in allofus.nih.gov). Additional and larger studies in the future should provide further insight into genetic contributions to CGN and also to its relationship with sexual orientation.

## Supplementary Information

Below is the link to the electronic supplementary material.

Supplementary file1 (DOCX 38555 kb)
