# Supplementary material for: Genome-Wide Linkage and Association Study of Childhood Gender Nonconformity in Males
Source: Arch Sex Behav. 2021 Sep 13;50(8):3377–83. doi: 10.1007/s10508-021-02146-x (PMC8604823; doi:10.1007/s10508-021-02146-x)
Supplement: Supplementary file 1 — Supplementary file1 (DOCX 38555 kb) [file 10508_2021_2146_MOESM1_ESM.docx]

**Supplementary Figure 1. Quantile-Quantile for GWAS of normCGN.**

**
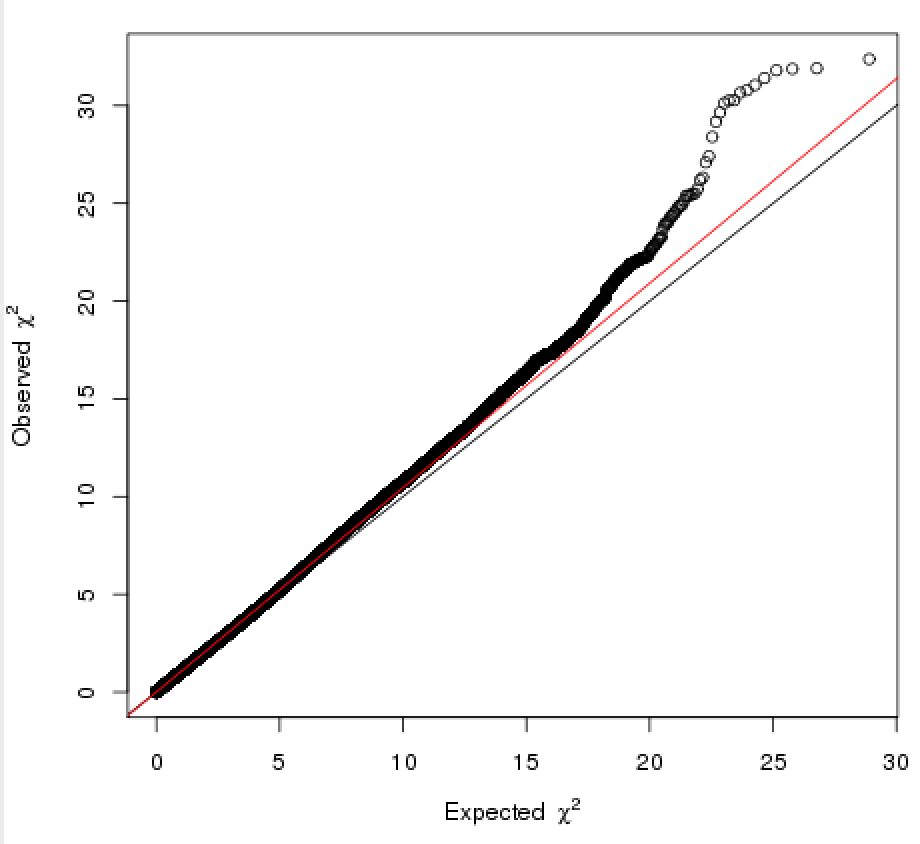
**

**Supplementary Figure 2. Regional association plot for chromosome 5.**


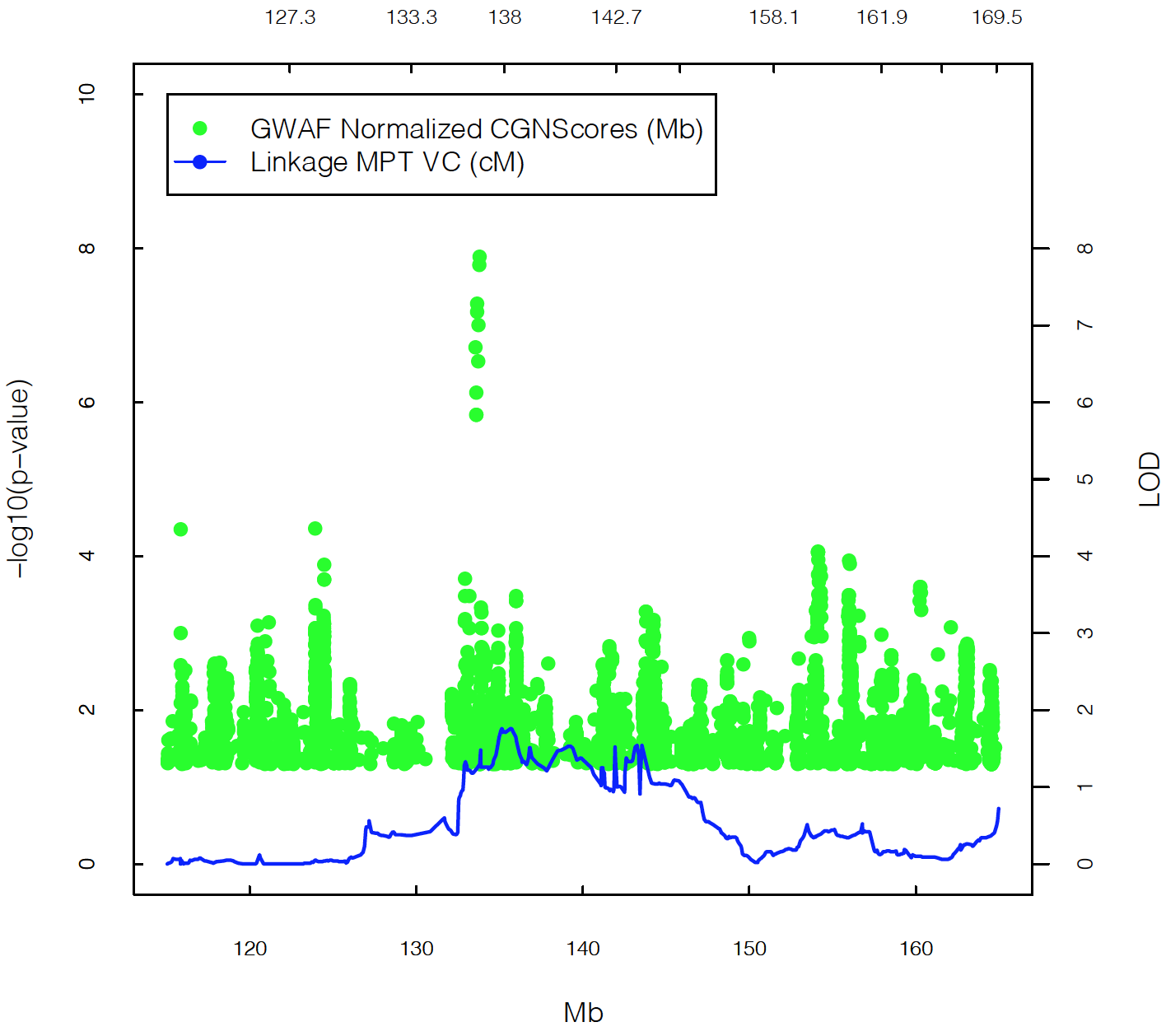


**Supplementary Figure 3. Regional association plot for chromosome 6.**


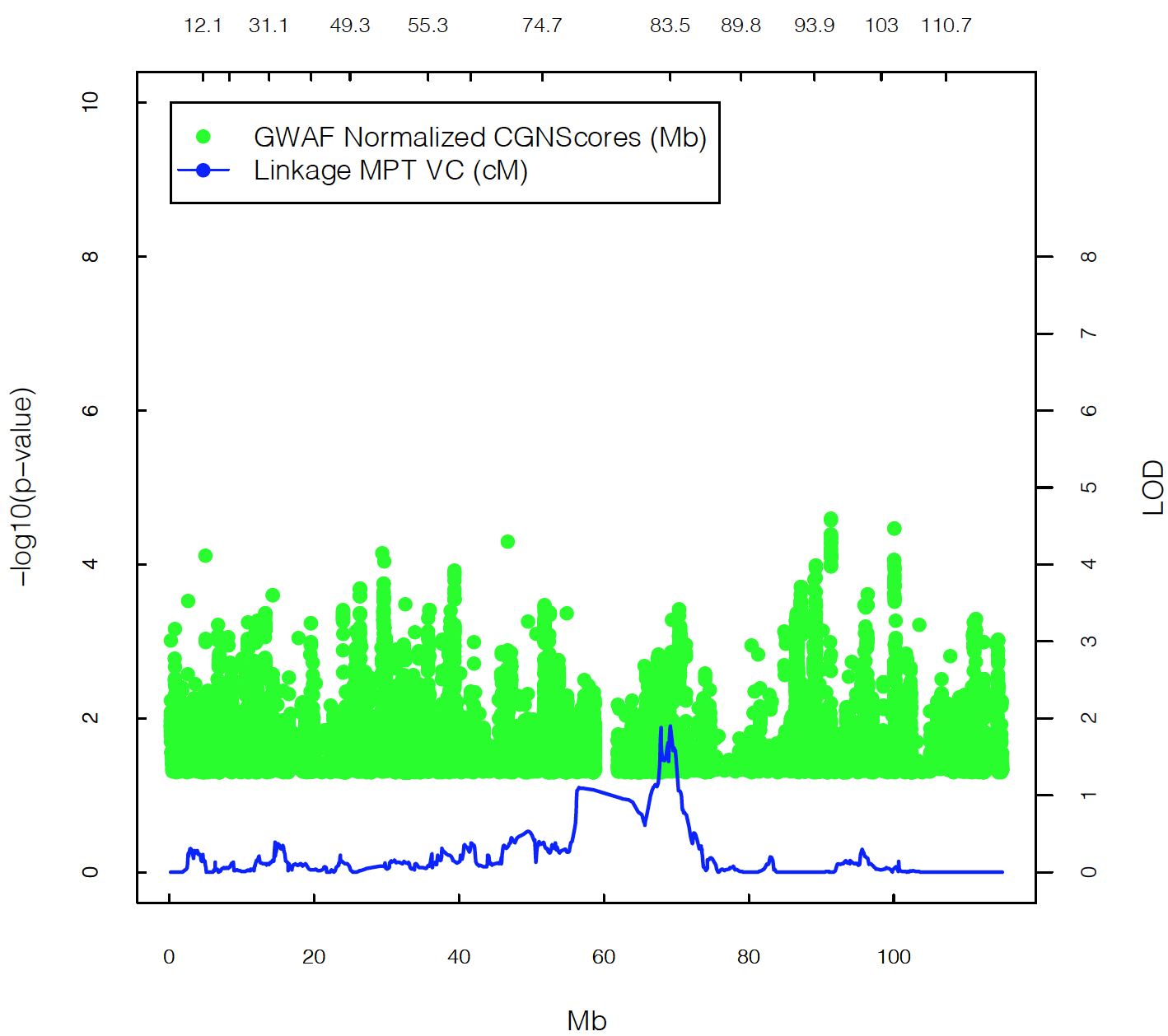


**Supplementary Figure 4. Regional association plot for chromosome 7.**


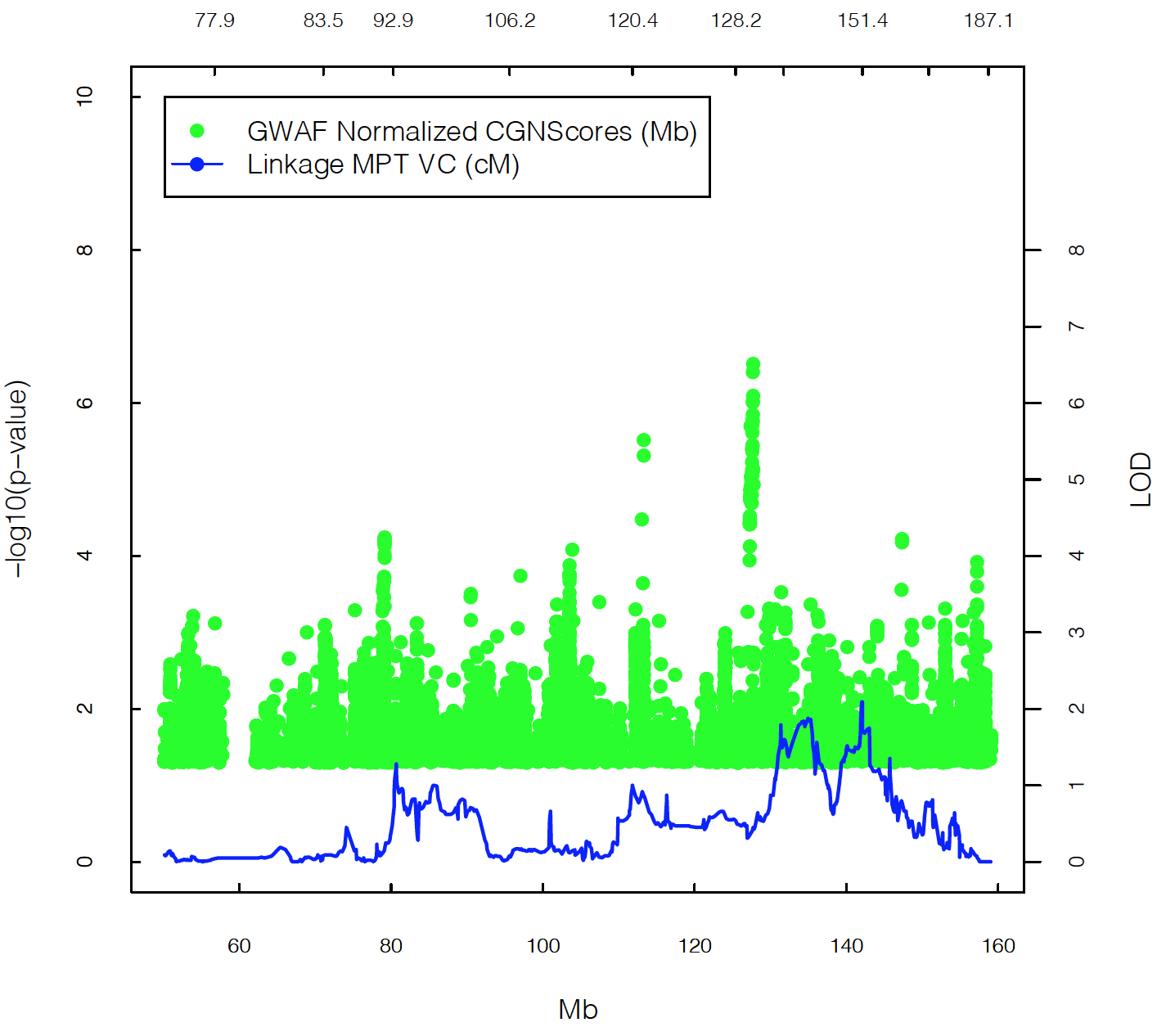


**Supplementary Figure 5. Regional association plot for chromosome 8.**


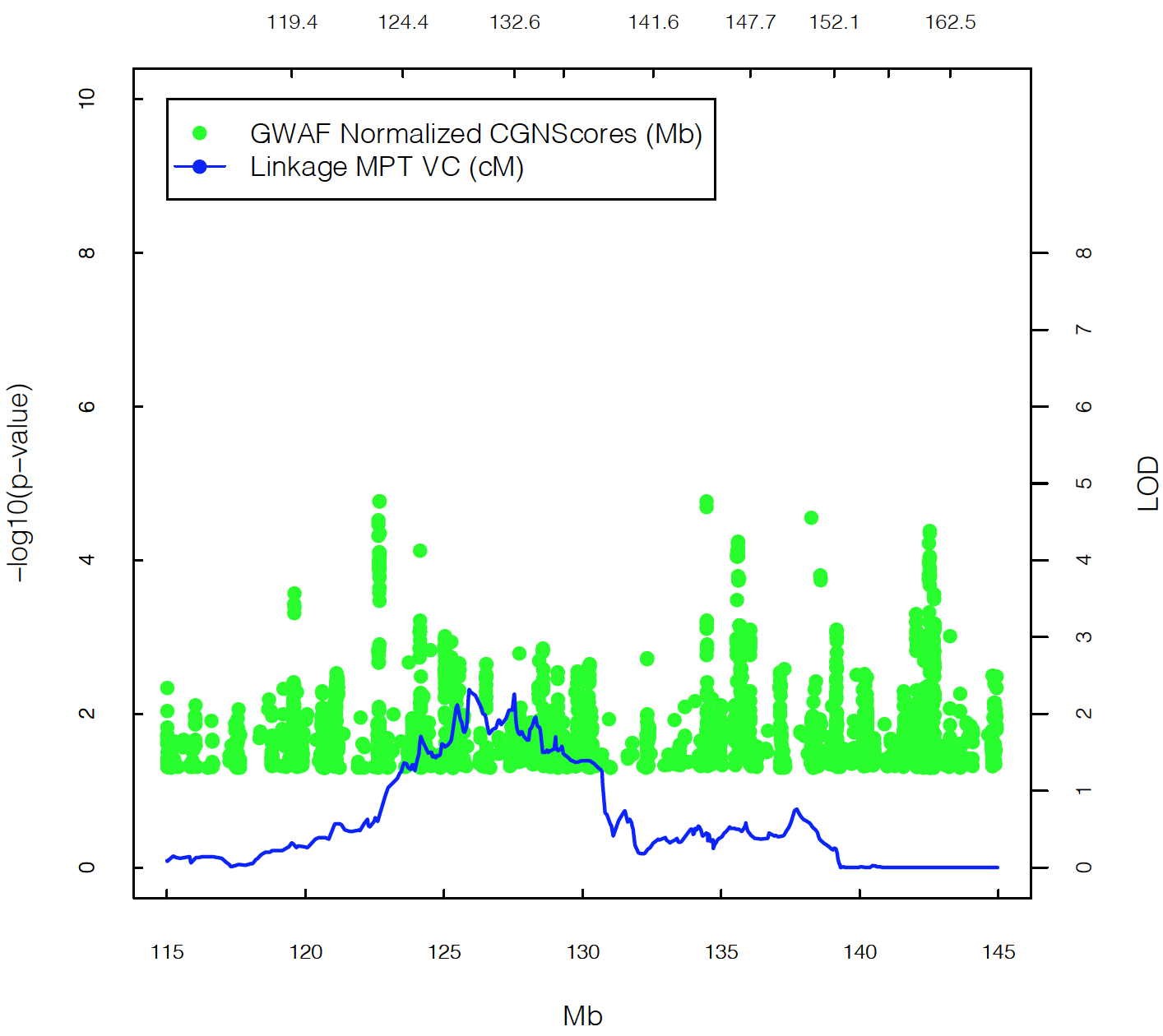


**Supplementary Figure 6. Regional association plot for chromosome 10.**


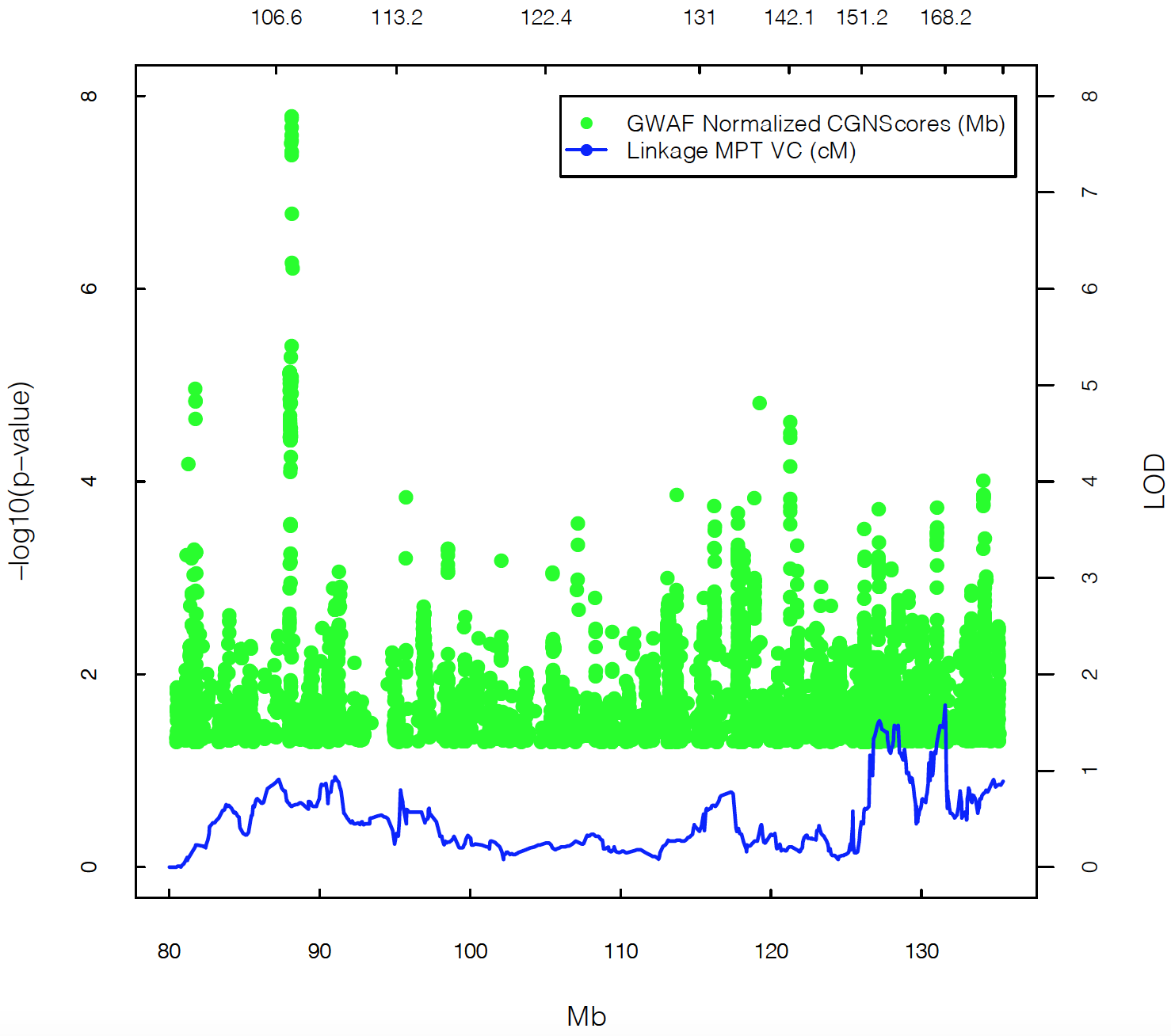


**Supplementary Figure 7. Linkage power estimates.**

**Supplementary Figure 8. Association power estimates.**

**Supplementary Table 1. Two-point linkage findings exceeding “suggestive” (LOD ≥ 2.2) linkage.**

| **Chr** | **SNP** | **bp** | **cM** | **LOD** |
| --- | --- | --- | --- | --- |
| 1 | rs693734 | 4,364,226 | 5.88 | 2.66 |
| 1 | rs351598 | 4,379,958 | 5.92 | 2.54 |
| 1 | rs619364 | 4,646,379 | 6.60 | 2.69 |
| 1 | rs17349719 | 5,001,809 | 7.67 | 2.51 |
| 1 | rs10915296 | 5,007,235 | 7.68 | 2.31 |
| 1 | rs536995 | 5,326,050 | 8.47 | 2.55 |
| 1 | rs548726 | 5,913,621 | 9.75 | 2.50 |
| 1 | rs11121090 | 8,168,634 | 13.89 | 2.64 |
| 1 | rs4418631 | 8,174,197 | 13.89 | 2.51 |
| 1 | rs9662290 | 9,362,341 | 15.46 | 2.21 |
| 1 | rs284267 | 10,656,351 | 16.93 | 2.46 |
| 1 | rs6429794 | 14,045,394 | 24.13 | 2.77 |
| 1 | rs16852866 | 14,053,474 | 24.14 | 2.73 |
| 1 | rs10489151 | 14,055,709 | 24.15 | 2.70 |
| 1 | rs1203682 | 14,080,682 | 24.19 | 3.42 |
| 1 | rs1980472 | 14,117,876 | 24.26 | 2.41 |
| 1 | rs7522358 | 14,223,723 | 24.46 | 2.25 |
| 1 | rs2744645 | 14,226,608 | 24.46 | 2.20 |
| 1 | rs12044299 | 14,288,119 | 24.58 | 2.26 |
| 1 | rs7522712 | 15,307,478 | 27.27 | 2.42 |
| 1 | rs10927613 | 15,308,798 | 27.27 | 2.42 |
| 1 | rs2473344 | 15,728,289 | 28.02 | 3.20 |
| 1 | rs2092324 | 15,733,394 | 28.03 | 2.79 |
| 1 | rs2977269 | 17,591,077 | 31.34 | 3.18 |
| 1 | rs2977303 | 17,611,548 | 31.38 | 2.87 |
| 1 | rs16862061 | 18,877,477 | 34.92 | 2.43 |
| 1 | rs10737452 | 21,199,764 | 39.60 | 2.30 |
| 1 | rs2744738 | 22,551,813 | 40.98 | 2.26 |
| 1 | rs6687818 | 24,901,998 | 44.18 | 2.33 |
| 1 | rs9328948 | 24,917,456 | 44.20 | 2.39 |
| 1 | rs16838019 | 29,540,532 | 47.56 | 2.35 |
| 1 | rs1111382 | 30,061,852 | 48.23 | 2.51 |
| 1 | rs894216 | 30,185,088 | 48.42 | 3.44 |
| 1 | rs11584521 | 30,215,072 | 48.48 | 2.93 |
| 1 | rs995197 | 30,215,680 | 48.48 | 2.83 |
| 1 | rs6425864 | 30,639,236 | 49.17 | 2.45 |
| 1 | rs7530575 | 30,639,318 | 49.17 | 3.38 |
| 1 | rs6704396 | 31,006,621 | 49.92 | 3.11 |
| 1 | rs12127272 | 31,007,037 | 49.92 | 3.04 |
| **1** | **rs555920** | **31,059,864** | **50.12** | **3.74** |
| 1 | rs6426044 | 31,077,028 | 50.19 | 2.23 |
| 1 | rs551060 | 34,179,408 | 53.81 | 2.20 |
| 1 | rs10914782 | 34,267,945 | 54.08 | 2.40 |
| 1 | rs1886637 | 34,269,435 | 54.08 | 2.47 |
| 1 | rs3845476 | 34,283,894 | 54.13 | 2.39 |
| 1 | rs3842834 | 34,284,118 | 54.13 | 2.39 |
| 1 | rs12067054 | 34,291,676 | 54.15 | 2.98 |
| 1 | rs3845477 | 34,291,836 | 54.15 | 2.98 |
| 1 | rs1950895 | 34,305,229 | 54.19 | 2.52 |
| 1 | rs1884981 | 34,306,177 | 54.19 | 3.08 |
| 1 | rs6694043 | 34,982,041 | 55.73 | 2.29 |
| 1 | rs6683126 | 38,681,026 | 60.85 | 2.29 |
| 1 | rs41499648 | 201,625,402 | 198.89 | 2.79 |
| 2 | rs11691477 | 17,463,724 | 38.70 | 3.57 |
| **2** | **rs877688** | **17,474,178** | **38.72** | **3.83** |
| 2 | rs7606821 | 18,057,168 | 39.66 | 2.21 |
| 2 | rs1384919 | 52,974,459 | 77.40 | 2.28 |
| 2 | rs7570229 | 75,866,236 | 100.39 | 2.28 |
| 2 | rs11894322 | 101,532,830 | 115.03 | 2.28 |
| 2 | rs4851507 | 102,495,917 | 115.69 | 2.39 |
| 2 | rs6543288 | 105,995,617 | 118.71 | 2.25 |
| 2 | rs980317 | 115,710,936 | 127.15 | 2.49 |
| 2 | rs17512698 | 118,755,669 | 128.89 | 2.54 |
| 2 | rs6719645 | 212,524,973 | 207.81 | 2.34 |
| 2 | rs998143 | 214,146,873 | 209.69 | 2.62 |
| 2 | rs6761272 | 223,660,306 | 225.99 | 2.38 |
| 2 | rs9653359 | 223,661,303 | 225.99 | 2.40 |
| 2 | rs6746278 | 228,621,558 | 231.48 | 2.51 |
| 3 | rs280791 | 1,647,955 | 3.90 | 2.46 |
| 3 | rs435995 | 1,655,790 | 3.92 | 2.38 |
| 3 | rs2648447 | 1,656,488 | 3.93 | 2.35 |
| 3 | rs2648449 | 1,656,864 | 3.93 | 2.36 |
| 3 | rs6789321 | 1,670,758 | 3.96 | 2.55 |
| 3 | rs7632130 | 1,671,088 | 3.96 | 2.59 |
| 3 | rs1384222 | 1,671,525 | 3.97 | 2.59 |
| 3 | rs4346532 | 1,671,618 | 3.97 | 2.69 |
| 3 | rs4519681 | 1,671,774 | 3.97 | 2.57 |
| 3 | rs5018408 | 1,672,678 | 3.97 | 2.56 |
| 3 | rs17019466 | 2,770,509 | 6.58 | 2.29 |
| 3 | rs6774407 | 3,673,983 | 11.22 | 2.70 |
| 3 | rs11130036 | 4,543,463 | 14.07 | 2.35 |
| 3 | rs9784254 | 4,571,005 | 14.14 | 2.84 |
| 3 | rs6784152 | 4,572,505 | 14.14 | 2.36 |
| 3 | rs7631197 | 4,994,869 | 15.26 | 2.51 |
| 3 | rs7635047 | 5,592,056 | 16.85 | 2.49 |
| 3 | rs4686377 | 9,877,452 | 27.32 | 2.46 |
| 3 | rs299647 | 12,539,238 | 30.51 | 2.30 |
| 3 | rs12635063 | 13,508,455 | 32.00 | 2.21 |
| 3 | rs905647 | 15,512,242 | 35.21 | 2.61 |
| 3 | rs336641 | 18,617,276 | 38.93 | 2.46 |
| 3 | rs404552 | 18,652,605 | 38.98 | 2.38 |
| 3 | rs6799759 | 18,700,071 | 39.04 | 2.25 |
| 3 | rs17204177 | 20,764,131 | 41.65 | 2.69 |
| 3 | rs1499004 | 20,880,872 | 41.80 | 2.51 |
| 3 | rs9829660 | 21,020,875 | 41.97 | 2.45 |
| 3 | rs1532129 | 21,667,698 | 42.79 | 2.22 |
| 3 | rs11919688 | 21,681,969 | 42.81 | 2.20 |
| 3 | rs7624502 | 22,244,794 | 43.46 | 2.36 |
| 3 | rs10510530 | 22,484,605 | 43.53 | 2.43 |
| 3 | rs1524317 | 22,746,444 | 43.72 | 2.33 |
| 3 | rs1402366 | 22,766,469 | 43.73 | 3.18 |
| 3 | rs696078 | 22,944,157 | 43.88 | 2.25 |
| 3 | rs7374732 | 23,203,454 | 44.29 | 2.25 |
| 3 | rs9831806 | 23,207,092 | 44.29 | 2.55 |
| 3 | rs11711194 | 23,648,116 | 44.99 | 2.71 |
| 3 | rs4858604 | 24,349,915 | 46.10 | 2.34 |
| 3 | rs9840442 | 25,080,041 | 47.28 | 2.53 |
| 3 | rs9848240 | 31,511,691 | 56.60 | 2.39 |
| 3 | rs6762236 | 32,159,162 | 57.52 | 2.48 |
| 3 | rs4955281 | 32,399,859 | 57.86 | 2.54 |
| 3 | rs11927331 | 32,937,008 | 58.63 | 2.44 |
| 3 | rs3752614 | 38,104,353 | 63.08 | 2.60 |
| 3 | rs6768315 | 38,109,571 | 63.09 | 2.57 |
| 3 | rs1417147 | 39,540,727 | 64.59 | 2.31 |
| 3 | rs552724 | 39,554,946 | 64.60 | 2.51 |
| 3 | rs816491 | 39,555,497 | 64.60 | 2.40 |
| 3 | rs864643 | 39,555,580 | 64.60 | 2.40 |
| 3 | rs1068953 | 39,556,561 | 64.60 | 2.60 |
| 3 | rs634338 | 39,564,243 | 64.61 | 2.50 |
| 3 | rs1631776 | 39,590,195 | 64.63 | 2.94 |
| 3 | rs9813883 | 146,287,491 | 153.13 | 2.22 |
| 3 | rs1431969 | 146,541,654 | 153.25 | 2.80 |
| 3 | rs7636940 | 146,544,813 | 153.25 | 2.89 |
| 3 | rs1521588 | 146,572,777 | 153.27 | 2.45 |
| 3 | rs1456135 | 150,648,997 | 159.39 | 2.69 |
| 3 | rs9822514 | 182,301,473 | 186.95 | 2.31 |
| 3 | rs9810603 | 184,986,743 | 191.42 | 2.48 |
| 3 | rs6444052 | 185,062,440 | 191.49 | 2.21 |
| 3 | rs7628096 | 186,087,169 | 193.88 | 3.22 |
| 3 | rs9878325 | 186,208,650 | 194.28 | 2.35 |
| 3 | rs12696575 | 187,217,274 | 197.82 | 2.61 |
| 3 | rs1523476 | 187,494,177 | 198.28 | 2.42 |
| 3 | rs9819305 | 187,757,553 | 199.42 | 2.33 |
| 3 | rs13092714 | 187,796,447 | 199.57 | 2.65 |
| 4 | rs2673414 | 13,053,092 | 26.15 | 2.36 |
| 4 | rs6821678 | 90,058,856 | 97.69 | 2.64 |
| 4 | rs362492 | 114,139,428 | 117.92 | 2.24 |
| 4 | rs12508397 | 114,309,283 | 118.15 | 2.31 |
| 4 | rs2165543 | 115,889,238 | 118.85 | 2.25 |
| 4 | rs1904480 | 115,961,723 | 118.88 | 2.26 |
| 4 | rs17031951 | 155,649,957 | 149.92 | 2.37 |
| 4 | rs10517602 | 155,653,240 | 149.92 | 2.37 |
| 4 | rs11133061 | 175,842,786 | 170.02 | 2.40 |
| 4 | rs4596220 | 175,865,542 | 170.02 | 2.48 |
| 5 | rs10472047 | 50,254,866 | 67.39 | 2.64 |
| 5 | rs6864527 | 62,494,598 | 76.94 | 2.49 |
| 5 | rs2126483 | 62,511,887 | 76.94 | 2.59 |
| 5 | rs1549249 | 64,455,558 | 77.88 | 2.35 |
| 5 | rs1705397 | 66,511,062 | 79.17 | 2.26 |
| 5 | rs245202 | 127,168,735 | 131.97 | 2.40 |
| 5 | rs25734 | 132,735,113 | 134.73 | 2.27 |
| 5 | rs7732903 | 136,227,992 | 138.72 | 2.97 |
| 5 | rs6861657 | 136,254,338 | 138.75 | 2.44 |
| 5 | rs6863244 | 136,335,948 | 138.82 | 2.92 |
| 5 | rs3813315 | 136,954,861 | 138.88 | 2.89 |
| 5 | rs4260664 | 136,962,286 | 138.88 | 3.38 |
| **5** | **rs2349010** | **136,969,235** | **138.88** | **4.28** |
| **5** | **rs11242393** | **136,971,101** | **138.88** | **4.28** |
| **5** | **rs2074349** | **136,972,660** | **138.88** | **4.26** |
| **5** | **rs13172798** | **136,990,675** | **138.88** | **4.28** |
| **5** | **rs17171566** | **136,996,297** | **138.88** | **4.45** |
| 5 | rs12653760 | 137,032,796 | 138.89 | 2.76 |
| 5 | rs4835672 | 137,568,867 | 138.93 | 2.34 |
| 5 | rs12656580 | 143,110,247 | 145.60 | 2.63 |
| 5 | rs917585 | 149,572,949 | 153.60 | 3.07 |
| 5 | rs1035396 | 151,864,264 | 158.21 | 2.22 |
| 5 | rs2878892 | 167,517,336 | 175.08 | 2.30 |
| 6 | rs6915338 | 3,035,523 | 8.09 | 2.33 |
| 6 | rs6920099 | 3,035,613 | 8.09 | 2.47 |
| 6 | rs11757974 | 56,138,377 | 79.88 | 2.24 |
| 6 | rs354391 | 66,319,480 | 81.63 | 2.78 |
| **6** | **rs4416661** | **67,688,283** | **82.73** | **3.64** |
| 6 | rs1083338 | 68,855,277 | 83.29 | 2.27 |
| 6 | rs2802710 | 69,321,495 | 83.56 | 2.26 |
| 7 | rs6977733 | 1,886,725 | 4.95 | 2.32 |
| 7 | rs10240036 | 2,486,368 | 5.83 | 2.81 |
| 7 | rs1554497 | 3,415,446 | 7.79 | 2.27 |
| 7 | rs1294640 | 7,378,904 | 13.36 | 2.93 |
| 7 | rs10952049 | 7,379,934 | 13.36 | 3.01 |
| 7 | rs9640039 | 7,407,152 | 13.38 | 3.02 |
| 7 | rs1294625 | 7,420,613 | 13.39 | 2.44 |
| 7 | rs12699238 | 11,610,592 | 22.56 | 2.21 |
| 7 | rs12699241 | 11,612,083 | 22.57 | 2.59 |
| 7 | rs6969041 | 80,953,663 | 93.67 | 2.29 |
| **7** | **rs2967925** | **81,046,460** | **93.81** | **3.80** |
| 7 | rs3801712 | 81,830,463 | 94.99 | 2.54 |
| 7 | rs17164334 | 88,410,013 | 99.92 | 2.42 |
| 7 | rs6975112 | 88,423,096 | 99.92 | 2.35 |
| 7 | rs6968172 | 88,448,121 | 99.93 | 2.52 |
| 7 | rs7796603 | 88,450,872 | 99.94 | 2.43 |
| 7 | rs4728798 | 88,736,883 | 100.19 | 2.66 |
| 7 | rs2709219 | 91,143,364 | 102.30 | 2.38 |
| 7 | rs6959147 | 96,369,360 | 106.55 | 2.48 |
| 7 | rs6943474 | 101,001,016 | 110.64 | 2.60 |
| 7 | rs6465799 | 101,007,557 | 110.65 | 2.29 |
| 7 | rs4729693 | 101,007,777 | 110.65 | 2.29 |
| 7 | rs7349984 | 113,102,425 | 121.03 | 2.45 |
| 7 | rs12176609 | 120,711,288 | 124.41 | 2.26 |
| 7 | rs6952251 | 122,547,130 | 126.21 | 2.35 |
| 7 | rs4319013 | 122,648,394 | 126.27 | 2.28 |
| 7 | rs7791660 | 123,129,020 | 126.55 | 2.33 |
| 7 | rs1833082 | 125,219,904 | 128.09 | 2.25 |
| 7 | rs11563684 | 126,853,171 | 128.86 | 2.55 |
| 7 | rs17340542 | 128,720,045 | 130.24 | 2.60 |
| 7 | rs13227095 | 128,723,943 | 130.25 | 2.81 |
| 7 | rs2129561 | 130,963,771 | 134.02 | 2.22 |
| 7 | rs4731794 | 131,008,336 | 134.10 | 2.37 |
| 7 | rs9656405 | 131,514,984 | 135.02 | 2.24 |
| 7 | rs1567857 | 131,727,515 | 135.41 | 2.55 |
| 7 | rs10954354 | 131,734,723 | 135.42 | 2.83 |
| 7 | rs9791411 | 131,744,656 | 135.44 | 2.27 |
| 7 | rs1514056 | 131,745,298 | 135.44 | 2.44 |
| 7 | rs156950 | 132,182,674 | 136.24 | 2.31 |
| 7 | rs1365364 | 132,276,066 | 136.41 | 2.65 |
| 7 | rs6948781 | 132,325,596 | 136.50 | 2.56 |
| 7 | rs6967574 | 132,560,155 | 136.93 | 2.27 |
| 7 | rs1862048 | 133,978,167 | 138.80 | 2.53 |
| 7 | rs1978504 | 134,006,933 | 138.81 | 2.66 |
| 7 | rs7779600 | 134,478,203 | 138.88 | 2.54 |
| 7 | rs3800752 | 134,630,642 | 138.96 | 3.09 |
| 7 | rs13224089 | 134,987,157 | 139.30 | 2.36 |
| 7 | rs13227663 | 136,340,237 | 141.32 | 2.38 |
| 7 | rs17506733 | 136,707,227 | 141.79 | 2.22 |
| 7 | rs11762896 | 136,709,168 | 141.79 | 2.33 |
| 7 | rs1424548 | 136,709,760 | 141.79 | 2.38 |
| 7 | rs11971309 | 136,712,155 | 141.79 | 2.46 |
| 7 | rs918974 | 136,770,849 | 141.87 | 2.20 |
| 7 | rs188915 | 136,962,095 | 142.11 | 2.73 |
| 7 | rs13247859 | 141,367,582 | 150.18 | 2.98 |
| 7 | rs17162409 | 141,369,007 | 150.18 | 2.92 |
| 7 | rs13225438 | 141,371,807 | 150.19 | 2.63 |
| 7 | rs4607527 | 141,728,635 | 150.83 | 2.93 |
| 7 | rs361467 | 142,223,967 | 151.42 | 2.39 |
| 7 | rs361466 | 142,224,011 | 151.42 | 2.39 |
| 7 | rs6950275 | 142,224,458 | 151.42 | 2.39 |
| 7 | rs6464492 | 142,224,915 | 151.42 | 2.41 |
| 7 | rs2734063 | 142,260,736 | 151.43 | 2.72 |
| 7 | rs2213187 | 142,267,510 | 151.43 | 2.43 |
| 8 | rs12679402 | 41,958,980 | 60.93 | 2.28 |
| 8 | rs7840827 | 41,969,770 | 60.93 | 2.53 |
| 8 | rs6468728 | 101,638,424 | 106.02 | 2.21 |
| 8 | rs7832270 | 101,642,870 | 106.03 | 2.46 |
| 8 | rs2514938 | 119,323,354 | 119.22 | 2.25 |
| 8 | rs16897107 | 123,534,675 | 124.38 | 2.32 |
| 8 | rs10086964 | 123,543,258 | 124.39 | 2.32 |
| 8 | rs10095188 | 123,833,865 | 125.50 | 2.81 |
| 8 | rs2385269 | 124,572,591 | 126.98 | 3.06 |
| 8 | rs6998986 | 124,887,024 | 127.53 | 2.24 |
| 8 | rs10956153 | 124,909,281 | 127.57 | 2.31 |
| 8 | rs12548510 | 124,923,123 | 127.59 | 2.42 |
| 8 | rs6981430 | 124,984,435 | 127.70 | 2.21 |
| 8 | rs3901290 | 125,702,761 | 128.76 | 2.59 |
| 8 | rs4871550 | 125,815,850 | 128.87 | 2.30 |
| 8 | rs16900177 | 126,024,314 | 129.07 | 2.68 |
| 8 | rs4601329 | 126,254,561 | 129.30 | 2.20 |
| 8 | rs4870946 | 126,603,194 | 129.81 | 2.76 |
| 8 | rs10086636 | 126,604,637 | 129.81 | 2.68 |
| 8 | rs6981560 | 126,607,466 | 129.81 | 2.89 |
| 8 | rs1454616 | 126,611,214 | 129.82 | 2.95 |
| 8 | rs1580096 | 126,689,385 | 129.94 | 2.21 |
| 8 | rs6985951 | 126,692,618 | 129.94 | 2.30 |
| 8 | rs780332 | 127,060,107 | 130.50 | 2.65 |
| 8 | rs780331 | 127,060,530 | 130.50 | 2.78 |
| 8 | rs780327 | 127,063,064 | 130.50 | 2.53 |
| 8 | rs2637834 | 127,068,262 | 130.51 | 3.16 |
| 8 | rs2721109 | 127,464,064 | 132.43 | 2.28 |
| 8 | rs4871714 | 127,528,089 | 132.61 | 2.26 |
| 8 | rs4870990 | 127,863,684 | 133.54 | 2.35 |
| 8 | rs979963 | 127,865,119 | 133.54 | 2.58 |
| 8 | rs7839993 | 127,871,473 | 133.56 | 2.71 |
| 8 | rs10956332 | 127,873,548 | 133.57 | 2.37 |
| 8 | rs6984837 | 127,901,649 | 133.64 | 2.63 |
| 8 | rs6470479 | 127,903,766 | 133.65 | 2.49 |
| 8 | rs4871004 | 127,982,089 | 133.87 | 2.76 |
| 8 | rs961023 | 128,005,150 | 133.93 | 2.97 |
| 8 | rs710885 | 128,026,128 | 133.99 | 2.94 |
| 8 | rs12056374 | 128,052,255 | 134.06 | 2.45 |
| 8 | rs1456310 | 128,052,433 | 134.06 | 2.55 |
| 8 | rs4288339 | 128,067,300 | 134.10 | 2.20 |
| 8 | rs6470505 | 128,255,861 | 134.63 | 2.55 |
| 8 | rs1962471 | 128,281,708 | 134.70 | 2.55 |
| 8 | rs1562430 | 128,387,852 | 134.99 | 2.22 |
| **8** | **rs7841264** | **128,466,814** | **135.21** | **3.67** |
| 8 | rs7004509 | 128,973,665 | 137.13 | 2.69 |
| 8 | rs759648 | 129,158,945 | 137.66 | 2.25 |
| 8 | rs13255672 | 129,171,082 | 137.69 | 2.95 |
| 8 | rs4733842 | 129,174,284 | 137.70 | 2.93 |
| 8 | rs4733598 | 129,181,647 | 137.72 | 3.11 |
| 8 | rs16902700 | 129,198,885 | 137.77 | 3.13 |
| 8 | rs7820652 | 129,215,374 | 137.82 | 2.91 |
| 8 | rs7010390 | 129,218,859 | 137.83 | 2.81 |
| 8 | rs4472475 | 130,222,035 | 138.87 | 2.73 |
| 8 | rs2553588 | 133,803,414 | 143.46 | 2.21 |
| 8 | rs235428 | 133,815,726 | 143.47 | 2.26 |
| 8 | rs180194 | 133,877,427 | 143.54 | 2.20 |
| 8 | rs864898 | 133,904,380 | 143.57 | 2.20 |
| 8 | rs2896714 | 134,495,614 | 145.52 | 2.40 |
| 9 | rs12343051 | 1,481,609 | 2.51 | 2.24 |
| 9 | rs1041506 | 9,022,772 | 19.85 | 2.81 |
| 9 | rs10511588 | 14,041,071 | 28.14 | 2.23 |
| 10 | rs1947567 | 88,018,949 | 107.21 | 2.90 |
| 10 | rs1934952 | 96,797,500 | 115.33 | 2.41 |
| 10 | rs529334 | 115,150,037 | 130.81 | 2.53 |
| 10 | rs1124096 | 115,156,226 | 130.82 | 2.93 |
| 10 | rs4460752 | 125,725,809 | 150.64 | 2.20 |
| 10 | rs10765137 | 129,388,646 | 159.56 | 2.20 |
| 10 | rs7901816 | 129,401,906 | 159.58 | 2.31 |
| 10 | rs1335010 | 130,035,410 | 161.00 | 2.27 |
| 10 | rs530466 | 131,427,717 | 167.73 | 2.47 |
| 10 | rs10764931 | 131,953,988 | 169.42 | 3.43 |
| 10 | rs10764936 | 131,984,387 | 169.51 | 3.40 |
| 10 | rs10764939 | 131,991,139 | 169.54 | 2.99 |
| 12 | rs3923482 | 8,024,362 | 22.21 | 2.62 |
| 12 | rs12227654 | 23,688,755 | 43.27 | 2.24 |
| 12 | rs11169567 | 51,204,938 | 64.11 | 2.36 |
| 12 | rs10846634 | 124,715,313 | 142.19 | 2.24 |
| 12 | rs1471164 | 128,938,990 | 156.54 | 2.28 |
| 13 | rs2589307 | 43,698,991 | 45.02 | 2.42 |
| 13 | rs9302001 | 95,463,392 | 86.78 | 2.73 |
| 13 | rs9521765 | 111,099,875 | 118.84 | 2.48 |
| 15 | rs1901721 | 37,060,580 | 35.50 | 2.75 |
| 15 | rs1520013 | 37,423,940 | 36.12 | 2.24 |
| 15 | rs2681314 | 37,424,430 | 36.12 | 2.72 |
| 15 | rs17622684 | 39,651,980 | 40.20 | 2.56 |
| 15 | rs8034217 | 40,721,700 | 42.51 | 3.18 |
| 15 | rs1906421 | 53,841,661 | 51.57 | 2.55 |
| 15 | rs573320 | 54,678,314 | 52.35 | 2.58 |
| 15 | rs11631518 | 56,441,360 | 53.68 | 2.25 |
| 15 | rs10518839 | 56,484,363 | 53.70 | 2.26 |
| 15 | rs12148258 | 57,955,128 | 54.67 | 3.37 |
| 15 | rs8024206 | 61,019,815 | 61.72 | 2.25 |
| 15 | rs11071673 | 62,819,584 | 64.77 | 2.36 |
| 15 | rs8030359 | 62,819,996 | 64.77 | 2.31 |
| 15 | rs2306102 | 70,135,095 | 73.66 | 2.27 |
| 15 | rs14726 | 77,400,607 | 80.70 | 2.44 |
| 15 | rs11072865 | 79,898,351 | 83.34 | 2.44 |
| 16 | rs11076893 | 5,255,874 | 12.57 | 2.31 |
| 16 | rs1882587 | 7,272,560 | 18.51 | 2.38 |
| 16 | rs7185684 | 7,281,941 | 18.54 | 2.28 |
| 16 | rs11077168 | 7,342,777 | 18.71 | 2.74 |
| 16 | rs9935078 | 8,604,798 | 23.25 | 2.31 |
| 16 | rs9925238 | 8,604,855 | 23.25 | 2.27 |
| 16 | rs6497738 | 10,253,636 | 25.23 | 2.22 |
| 16 | rs9939762 | 77,640,970 | 93.43 | 2.60 |
| 17 | rs205052 | 11,286,954 | 31.97 | 2.30 |
| 17 | rs1034899 | 11,306,660 | 32.00 | 2.26 |
| 17 | rs8182326 | 13,091,483 | 36.78 | 2.62 |
| 18 | rs565151 | 356,095 | 0.60 | 2.46 |
| 18 | rs4799499 | 36,712,399 | 59.98 | 2.24 |
| 18 | rs4800083 | 36,719,751 | 59.98 | 2.20 |
| 18 | rs4239421 | 36,755,757 | 59.99 | 2.42 |
| 18 | rs8094653 | 37,686,961 | 60.44 | 2.42 |
| 18 | rs487386 | 44,377,004 | 66.38 | 2.50 |
| 18 | rs8096263 | 44,515,216 | 66.58 | 2.51 |
| 18 | rs1319945 | 46,340,843 | 69.77 | 2.24 |
| 18 | rs12458118 | 53,276,116 | 76.37 | 2.62 |
| 18 | rs9946669 | 53,311,559 | 76.39 | 2.34 |
| 18 | rs1382117 | 53,461,945 | 76.57 | 2.23 |
| 18 | rs644016 | 55,164,547 | 78.37 | 2.39 |
| 18 | rs2019535 | 55,444,738 | 78.85 | 2.34 |
| 18 | rs7226831 | 67,847,005 | 98.02 | 2.33 |
| 18 | rs2282574 | 74,738,305 | 114.20 | 2.35 |
| 18 | rs8084836 | 76,647,841 | 119.91 | 2.72 |
| 18 | rs12458828 | 76,648,522 | 119.91 | 2.71 |
| 18 | rs12607938 | 76,651,887 | 119.92 | 2.67 |
| 18 | rs3786228 | 77,464,170 | 121.29 | 2.26 |
| 18 | rs2007483 | 77,476,131 | 121.30 | 2.41 |
| 19 | rs12980856 | 5,513,059 | 18.04 | 2.49 |
| 19 | rs11084178 | 53,212,361 | 92.78 | 2.26 |
| 19 | rs10853854 | 53,232,128 | 92.83 | 2.37 |
| 19 | rs583569 | 54,263,104 | 97.97 | 2.84 |
| 20 | rs6041046 | 12,025,424 | 35.30 | 2.34 |
| 20 | rs6049828 | 24,627,964 | 54.35 | 2.32 |
| 20 | rs6015857 | 59,412,752 | 104.71 | 2.81 |
| 20 | rs6015858 | 59,413,423 | 104.71 | 2.20 |
| 21 | rs11702385 | 18,541,509 | 11.69 | 2.26 |
| 21 | rs8131431 | 19,134,859 | 12.61 | 2.56 |
| 21 | rs2834844 | 36,581,730 | 41.73 | 2.98 |
| 21 | rs1033335 | 36,585,069 | 41.74 | 2.45 |
| 21 | rs7282109 | 37,771,010 | 42.90 | 2.89 |
| 22 | rs5750578 | 22,490,837 | 14.90 | 2.27 |
| 23 | rs5961914 | 5,912,923 | 12.77 | 2.29 |

^a^ Abbreviations include: bp (base pair), cM (centimorgan), and LOD (two-point LOD score). Note: Base pair location per Human Feb. 2009 (GRCh37/hg19); and cM is for deCODE genetic map. Rows with SNPs with “significant” two-point linkage (LOD ≥ 3.6) (Lander & Kruglyak, 1995) are bolded.

**Supplementary Table 2. Regions on chromosomes with SNPs associated at *p*< 10^-5^.**

| Chr | bp | SNP | EA | RA | EAF | info | beta | se | *p*val |
| --- | --- | --- | --- | --- | --- | --- | --- | --- | --- |
| 1 | 54,917,533 | rs72665995 | G | A | 0.904 | 0.746 | -0.100 | 0.022 | 4.67E-06 |
| 1 | 59,194,996 | rs201030007 | ATAT | A | 0.897 | 0.769 | -0.083 | 0.018 | 4.04E-06 |
| 1 | 59,205,102 | rs1969772 | G | A | 0.718 | 1.000 | -0.055 | 0.012 | 9.94E-06 |
| 1 | 168,663,274 | rs17502733 | C | T | 0.948 | 1.000 | -0.100 | 0.022 | 7.10E-06 |
| 2 | 28,618,161 | rs12473028 | G | A | 0.579 | 0.852 | 0.053 | 0.011 | 3.14E-06 |
| 2 | 79,218,464 | rs10204507 | C | T | 0.947 | 0.739 | 0.129 | 0.028 | 3.68E-06 |
| 4 | 65,823,213 | rs1561643 | T | C | 0.795 | 0.726 | -0.079 | 0.016 | 1.52E-06 |
| 5 | 36,723,323 | rs36652 | A | G | 0.897 | 1.000 | 0.084 | 0.017 | 4.66E-07 |
| 5 | 36,723,395 | rs36651 | C | T | 0.897 | 1.000 | 0.084 | 0.017 | 4.66E-07 |
| 5 | 36,723,848 | rs3832338 | T | TA | 0.897 | 0.994 | 0.084 | 0.017 | 4.45E-07 |
| 5 | 36,735,284 | rs42914 | C | A | 0.892 | 0.982 | 0.079 | 0.017 | 1.67E-06 |
| 5 | 57,577,692 | rs4700272 | G | A | 0.830 | 0.987 | 0.072 | 0.016 | 4.16E-06 |
| 5 | 57,583,930 | rs62366747 | C | T | 0.830 | 0.989 | 0.072 | 0.016 | 4.38E-06 |
| 5 | 57,587,004 | rs7380022 | A | G | 0.834 | 0.980 | 0.074 | 0.016 | 2.85E-06 |
| 5 | 57,587,561 | rs10077200 | T | A | 0.830 | 0.992 | 0.071 | 0.016 | 4.77E-06 |
| 5 | 57,591,195 | rs62366314 | G | A | 0.830 | 0.994 | 0.071 | 0.016 | 5.02E-06 |
| 5 | 57,596,070 | rs4700274 | A | T | 0.829 | 0.997 | 0.069 | 0.016 | 8.67E-06 |
| 5 | 57,596,330 | rs6871438 | T | C | 0.829 | 0.996 | 0.069 | 0.015 | 8.76E-06 |
| 5 | 133,557,562 | rs113707589 | T | C | 0.941 | 0.757 | -0.130 | 0.025 | 1.94E-07 |
| 5 | 133,605,943 | rs199879362 | TA | T | 0.947 | 0.767 | -0.131 | 0.027 | 7.52E-07 |
| 5 | 133,605,944 | rs10479072 | A | T | 0.945 | 0.755 | -0.128 | 0.026 | 1.46E-06 |
| **5** | **133,646,354** | **rs13157092** | **T** | **G** | **0.941** | **0.782** | **-0.137** | **0.025** | **6.62E-08** |
| **5** | **133,650,969** | **rs201102575** | **G** | **GT** | **0.938** | **0.781** | **-0.132** | **0.024** | **5.20E-08** |
| 5 | 133,717,987 | rs35911965 | C | CA | 0.938 | 0.767 | -0.129 | 0.025 | 2.89E-07 |
| 5 | 133,735,557 | rs143445950 | C | A | 0.943 | 0.762 | -0.142 | 0.027 | 9.88E-08 |
| 5 | 133,793,417 | rs17518213 | G | A | 0.948 | 0.819 | -0.142 | 0.025 | 1.64E-08 |
| 5 | 133,807,035 | rs113946051 | G | A | 0.947 | 0.833 | -0.141 | 0.025 | 1.27E-08 |
| 7 | 113,280,097 | rs2396670 | G | A | 0.598 | 0.888 | 0.064 | 0.014 | 4.81E-06 |
| 7 | 113,281,543 | rs2396671 | G | A | 0.612 | 0.893 | 0.065 | 0.014 | 3.05E-06 |
| 7 | 127,375,944 | rs111361464 | A | C | 0.857 | 0.968 | -0.079 | 0.017 | 2.00E-06 |
| 7 | 127,392,094 | rs73234897 | A | G | 0.857 | 0.968 | -0.079 | 0.017 | 1.98E-06 |
| 7 | 127,413,555 | rs717944 | T | C | 0.874 | 0.985 | -0.079 | 0.018 | 9.13E-06 |
| 7 | 127,556,713 | rs1075206 | T | C | 0.870 | 0.995 | 0.077 | 0.017 | 5.86E-06 |
| 7 | 127,562,465 | rs759056 | G | A | 0.869 | 1.000 | 0.077 | 0.017 | 3.99E-06 |
| 7 | 127,584,608 | rs917831 | T | A | 0.855 | 0.998 | 0.070 | 0.016 | 8.19E-06 |
| 7 | 127,586,086 | rs6948493 | A | G | 0.870 | 1.000 | 0.079 | 0.017 | 2.46E-06 |
| 7 | 127,587,046 | rs58040224 | A | AC | 0.855 | 0.999 | 0.070 | 0.016 | 8.16E-06 |
| 7 | 127,589,753 | rs887745 | T | A | 0.855 | 0.999 | 0.070 | 0.016 | 8.06E-06 |
| 7 | 127,592,643 | rs4731387 | T | C | 0.855 | 0.999 | 0.070 | 0.016 | 7.65E-06 |
| 7 | 127,592,643 | rs4731387 | T | C | 0.855 | 0.999 | 0.070 | 0.016 | 7.65E-06 |
| 7 | 127,594,068 | rs3808073 | T | A | 0.855 | 0.998 | 0.070 | 0.016 | 7.38E-06 |
| 7 | 127,594,304 | rs6963614 | C | T | 0.869 | 0.997 | 0.075 | 0.017 | 8.73E-06 |
| 7 | 127,601,254 | rs1362267 | C | T | 0.855 | 0.996 | 0.072 | 0.016 | 4.24E-06 |
| 7 | 127,607,474 | rs720300 | A | G | 0.869 | 0.997 | -0.080 | 0.017 | 1.86E-06 |
| 7 | 127,612,009 | rs1193334 | G | A | 0.869 | 0.997 | -0.080 | 0.017 | 1.92E-06 |
| 7 | 127,619,502 | rs1193337 | C | A | 0.869 | 0.998 | -0.080 | 0.017 | 1.78E-06 |
| 7 | 127,620,972 | rs3214415 | TA | T | 0.854 | 0.998 | -0.073 | 0.016 | 3.74E-06 |
| 7 | 127,621,738 | rs17676908 | T | G | 0.869 | 0.998 | -0.080 | 0.017 | 1.73E-06 |
| 7 | 127,636,958 | rs17676986 | C | T | 0.854 | 0.999 | -0.073 | 0.016 | 3.55E-06 |
| 7 | 127,638,703 | rs1362897 | G | A | 0.869 | 1.000 | -0.080 | 0.017 | 1.65E-06 |
| 7 | 127,657,793 | rs17733988 | G | A | 0.869 | 0.997 | -0.081 | 0.017 | 1.41E-06 |
| 7 | 127,668,071 | rs3808058 | C | T | 0.872 | 0.992 | -0.087 | 0.017 | 3.93E-07 |
| 7 | 127,669,584 | rs73238074 | T | C | 0.858 | 0.994 | -0.078 | 0.016 | 9.82E-07 |
| 7 | 127,680,693 | rs3808057 | T | C | 0.858 | 0.993 | -0.078 | 0.016 | 9.56E-07 |
| 7 | 127,694,032 | rs79436018 | T | C | 0.881 | 0.960 | -0.089 | 0.018 | 8.05E-07 |
| 7 | 127,695,857 | rs3757757 | G | A | 0.873 | 0.992 | -0.087 | 0.017 | 3.10E-07 |
| 7 | 127,717,435 | rs3808053 | T | G | 0.903 | 0.983 | -0.092 | 0.020 | 7.39E-06 |
| 7 | 127,718,888 | rs73238099 | C | G | 0.903 | 0.982 | -0.092 | 0.020 | 7.36E-06 |
| 8 | 16,794,434 | rs969424 | G | C | 0.873 | 0.922 | -0.073 | 0.016 | 8.47E-06 |
| 8 | 16,795,157 | rs970250 | C | T | 0.945 | 0.868 | 0.099 | 0.021 | 2.63E-06 |
| 8 | 16,795,342 | rs113238080 | C | T | 0.945 | 0.869 | 0.099 | 0.021 | 2.65E-06 |
| 8 | 16,796,669 | rs1602904 | C | T | 0.944 | 0.877 | 0.098 | 0.021 | 2.77E-06 |
| 8 | 16,796,774 | rs75366107 | G | A | 0.944 | 0.877 | 0.099 | 0.021 | 2.76E-06 |
| 8 | 16,796,967 | rs145105144 | G | A | 0.943 | 0.873 | 0.094 | 0.021 | 8.44E-06 |
| 8 | 16,798,143 | rs74421127 | C | T | 0.945 | 0.877 | 0.099 | 0.021 | 2.62E-06 |
| 8 | 16,798,317 | rs76011375 | G | A | 0.945 | 0.877 | 0.099 | 0.021 | 2.59E-06 |
| 8 | 16,798,590 | rs79882714 | G | A | 0.945 | 0.877 | 0.099 | 0.021 | 2.57E-06 |
| 8 | 16,799,623 | rs1094730 | A | G | 0.945 | 0.876 | -0.098 | 0.021 | 2.94E-06 |
| 8 | 16,799,981 | rs143332454 | A | ACT | 0.945 | 0.878 | 0.099 | 0.021 | 2.46E-06 |
| 8 | 16,800,480 | rs1608492 | T | A | 0.945 | 0.878 | 0.099 | 0.021 | 2.44E-06 |
| 8 | 16,800,703 | rs1608491 | T | C | 0.945 | 0.878 | 0.099 | 0.021 | 2.43E-06 |
| 8 | 16,807,678 | rs77066865 | A | G | 0.945 | 0.882 | 0.099 | 0.021 | 2.18E-06 |
| 8 | 16,808,752 | rs117801885 | G | A | 0.945 | 0.881 | 0.098 | 0.021 | 2.87E-06 |
| 8 | 16,811,048 | rs1586963 | G | C | 0.945 | 0.888 | 0.096 | 0.021 | 5.30E-06 |
| 8 | 16,814,287 | rs4922247 | T | C | 0.946 | 0.891 | 0.096 | 0.021 | 5.20E-06 |
| 8 | 16,816,092 | rs17488597 | C | T | 0.946 | 0.892 | 0.096 | 0.021 | 5.21E-06 |
| 8 | 16,822,735 | rs111656527 | A | G | 0.946 | 0.895 | 0.095 | 0.021 | 5.42E-06 |
| 8 | 16,826,151 | rs77921566 | A | G | 0.946 | 0.898 | 0.095 | 0.021 | 5.58E-06 |
| 8 | 16,827,952 | rs113214464 | C | T | 0.946 | 0.898 | 0.095 | 0.021 | 5.60E-06 |
| 8 | 16,829,415 | rs188643130 | C | T | 0.946 | 0.898 | 0.095 | 0.021 | 5.62E-06 |
| 8 | 16,830,251 | rs77027839 | C | T | 0.946 | 0.900 | 0.095 | 0.021 | 5.75E-06 |
| 8 | 16,831,373 | rs78668508 | T | A | 0.945 | 0.898 | 0.096 | 0.021 | 4.49E-06 |
| 8 | 16,831,749 | rs145671048 | A | C | 0.943 | 0.883 | 0.093 | 0.020 | 5.32E-06 |
| 8 | 16,834,712 | rs146535975 | G | C | 0.947 | 0.900 | 0.096 | 0.021 | 4.26E-06 |
| 8 | 16,835,064 | rs138865200 | C | T | 0.947 | 0.899 | 0.097 | 0.021 | 4.11E-06 |
| 8 | 16,836,151 | rs147066342 | C | T | 0.947 | 0.897 | 0.097 | 0.021 | 3.68E-06 |
| 8 | 16,836,166 | rs147817105 | C | A | 0.947 | 0.897 | 0.097 | 0.021 | 3.68E-06 |
| 8 | 16,836,295 | rs117774283 | A | T | 0.947 | 0.897 | 0.097 | 0.021 | 3.64E-06 |
| 8 | 16,836,558 | rs139653034 | A | G | 0.949 | 0.875 | 0.099 | 0.022 | 4.58E-06 |
| 8 | 16,836,619 | rs142964395 | T | C | 0.949 | 0.874 | 0.099 | 0.022 | 4.58E-06 |
| 8 | 16,836,931 | rs55779133 | C | A | 0.947 | 0.895 | 0.097 | 0.021 | 3.41E-06 |
| 8 | 16,837,079 | rs56156413 | G | A | 0.947 | 0.895 | 0.098 | 0.021 | 3.36E-06 |
| 8 | 16,837,268 | rs55824897 | G | T | 0.947 | 0.895 | 0.098 | 0.021 | 3.33E-06 |
| 8 | 16,837,362 | rs55666566 | A | G | 0.947 | 0.894 | 0.098 | 0.021 | 3.29E-06 |
| 8 | 16,837,501 | rs139734595 | G | GGAAAAGC | 0.947 | 0.894 | 0.098 | 0.021 | 3.25E-06 |
| 8 | 16,838,941 | rs79038165 | T | C | 0.947 | 0.892 | 0.098 | 0.021 | 2.90E-06 |
| 8 | 16,839,011 | rs17488730 | C | G | 0.948 | 0.888 | 0.102 | 0.021 | 1.15E-06 |
| 8 | 16,839,170 | rs78010178 | A | T | 0.947 | 0.891 | 0.099 | 0.021 | 2.85E-06 |
| 8 | 16,839,195 | rs75404687 | G | A | 0.947 | 0.891 | 0.099 | 0.021 | 2.86E-06 |
| 8 | 16,839,403 | rs74623563 | C | A | 0.947 | 0.891 | 0.099 | 0.021 | 2.81E-06 |
| 8 | 16,839,465 | rs77834600 | G | A | 0.947 | 0.891 | 0.099 | 0.021 | 2.81E-06 |
| 8 | 16,839,634 | rs112721448 | G | T | 0.947 | 0.888 | 0.098 | 0.021 | 3.31E-06 |
| 8 | 16,840,697 | rs17488758 | A | G | 0.947 | 0.889 | 0.099 | 0.021 | 2.57E-06 |
| 8 | 16,840,799 | rs2177413 | A | T | 0.947 | 0.889 | 0.099 | 0.021 | 2.53E-06 |
| 8 | 16,841,217 | rs950550 | C | T | 0.947 | 0.888 | 0.100 | 0.021 | 2.43E-06 |
| 8 | 16,841,497 | rs4387015 | A | T | 0.947 | 0.886 | 0.100 | 0.021 | 2.33E-06 |
| 8 | 16,845,517 | rs77919434 | G | A | 0.950 | 0.820 | 0.115 | 0.024 | 1.43E-06 |
| 9 | 1,450,274 | rs7037453 | C | A | 0.774 | 1.000 | -0.059 | 0.013 | 9.71E-06 |
| 10 | 88,009,253 | rs117789378 | G | A | 0.949 | 0.942 | -0.104 | 0.023 | 7.45E-06 |
| 10 | 88,010,217 | rs117824019 | G | A | 0.949 | 0.950 | -0.104 | 0.023 | 7.72E-06 |
| 10 | 88,010,576 | rs141303046 | C | T | 0.949 | 0.951 | -0.104 | 0.023 | 7.67E-06 |
| 10 | 88,012,514 | rs79580489 | C | T | 0.949 | 0.954 | -0.103 | 0.023 | 7.55E-06 |
| 10 | 88,013,396 | rs118113965 | G | A | 0.949 | 0.956 | -0.103 | 0.023 | 7.55E-06 |
| 10 | 88,014,980 | rs117470379 | C | G | 0.949 | 0.959 | -0.103 | 0.023 | 7.44E-06 |
| 10 | 88,016,840 | rs1870154 | C | T | 0.949 | 0.962 | -0.103 | 0.023 | 7.32E-06 |
| 10 | 88,017,150 | rs1870152 | T | G | 0.949 | 0.963 | -0.103 | 0.023 | 7.30E-06 |
| 10 | 88,018,014 | rs116850501 | T | A | 0.949 | 0.964 | -0.103 | 0.023 | 7.21E-06 |
| 10 | 88,021,322 | rs79830270 | A | C | 0.949 | 0.965 | -0.102 | 0.023 | 8.63E-06 |
| 10 | 88,022,276 | rs1870159 | C | A | 0.949 | 0.965 | -0.102 | 0.023 | 9.45E-06 |
| 10 | 88,073,338 | rs1896525 | A | G | 0.937 | 0.986 | 0.092 | 0.021 | 9.82E-06 |
| 10 | 88,096,204 | rs4934187 | A | G | 0.948 | 0.936 | 0.097 | 0.021 | 5.04E-06 |
| **10** | **88,097,538** | **rs10788491** | **C** | **T** | **0.949** | **0.918** | **0.115** | **0.021** | **3.09E-08** |
| **10** | **88,134,529** | **rs10430710** | **G** | **T** | **0.948** | **0.924** | **0.119** | **0.021** | **2.89E-08** |
| **10** | **88,134,752** | **rs10430711** | **C** | **G** | **0.948** | **0.925** | **0.120** | **0.021** | **2.09E-08** |
| **10** | **88,136,186** | **rs7900992** | **T** | **C** | **0.948** | **0.927** | **0.119** | **0.021** | **2.52E-08** |
| **10** | **88,137,225** | **rs4933393** | **G** | **A** | **0.948** | **0.927** | **0.117** | **0.021** | **3.76E-08** |
| **10** | **88,137,299** | **rs4934195** | **C** | **T** | **0.948** | **0.927** | **0.117** | **0.021** | **3.79E-08** |
| **10** | **88,138,165** | **rs1008912** | **C** | **T** | **0.948** | **0.931** | **0.117** | **0.021** | **1.62E-08** |
| **10** | **88,138,515** | **rs10788498** | **T** | **C** | **0.948** | **0.930** | **0.116** | **0.021** | **1.71E-08** |
| **10** | **88,138,813** | **rs7913719** | **T** | **C** | **0.950** | **0.938** | **0.114** | **0.021** | **4.05E-08** |
| 10 | 88,140,109 | rs4934196 | C | A | 0.947 | 0.939 | 0.100 | 0.022 | 8.16E-06 |
| 10 | 88,140,625 | rs11202010 | C | T | 0.946 | 0.937 | 0.102 | 0.022 | 3.85E-06 |
| 10 | 88,141,331 | rs1863827 | T | C | 0.947 | 0.943 | 0.099 | 0.022 | 8.92E-06 |
| 10 | 88,153,925 | rs1863820 | T | C | 0.950 | 0.953 | 0.109 | 0.021 | 1.65E-07 |
| 10 | 88,163,596 | rs61859068 | A | G | 0.920 | 0.779 | 0.102 | 0.020 | 5.31E-07 |
| 10 | 88,203,208 | rs7094269 | T | C | 0.945 | 0.904 | 0.102 | 0.020 | 6.06E-07 |
| 11 | 19,866,917 | rs894555 | T | C | 0.897 | 0.779 | -0.082 | 0.018 | 5.59E-06 |
| 11 | 19,871,230 | rs1838055 | G | C | 0.899 | 0.809 | -0.083 | 0.018 | 5.46E-06 |
| 11 | 19,871,312 | rs2165802 | T | C | 0.899 | 0.809 | -0.083 | 0.018 | 5.49E-06 |
| 11 | 19,871,905 | rs12361438 | A | G | 0.893 | 0.808 | -0.081 | 0.018 | 4.12E-06 |
| 11 | 19,879,351 | rs12361114 | T | C | 0.894 | 0.803 | -0.079 | 0.018 | 8.08E-06 |
| 11 | 33,638,940 | rs117890846 | A | G | 0.949 | 0.701 | -0.135 | 0.027 | 6.24E-07 |
| 11 | 66,464,561 | rs114901285 | A | T | 0.940 | 0.953 | -0.090 | 0.019 | 1.05E-06 |
| 11 | 66,711,195 | rs117905790 | C | A | 0.885 | 0.984 | -0.071 | 0.016 | 9.71E-06 |
| 11 | 116,375,253 | rs7107783 | A | G | 0.539 | 0.642 | -0.062 | 0.014 | 7.94E-06 |
| 11 | 134,502,285 | rs3019642 | G | A | 0.684 | 0.975 | -0.055 | 0.012 | 6.79E-06 |
| 11 | 134,503,875 | rs3019644 | C | T | 0.685 | 0.982 | -0.055 | 0.012 | 6.54E-06 |
| 12 | 47,434,141 | rs17097518 | A | G | 0.919 | 0.852 | -0.091 | 0.020 | 8.21E-06 |
| 13 | 112,250,415 | rs77197064 | G | A | 0.942 | 0.691 | 0.116 | 0.025 | 2.75E-06 |
| 18 | 76,530,270 | rs72992848 | G | A | 0.948 | 0.635 | -0.117 | 0.025 | 3.60E-06 |
| 21 | 37,999,053 | rs2091854 | G | C | 0.915 | 0.816 | -0.086 | 0.019 | 4.39E-06 |
| 21 | 42,866,107 | rs2838042 | T | C | 0.767 | 0.679 | -0.074 | 0.015 | 5.98E-07 |

^a^ Abbreviations include: EA (effect allele), RA (reference allele), and EAF (effect-allele frequency). Note: Base pair location per Human Feb. 2009 (GRCh37/hg19). Rows with SNPs with genome-wide significant association (*p* < 5 × 10^-8^) are bolded.

**Supplementary Text.**

**Statistical Power.**

We used Genetic Power Calculator (Purcell, Cherny, & Sham, 2003) to estimate power for linkage and association tests in 372 families, which is the number of independent families used in the final CGN analyses. For simplicity, we assumed only sibling pairs with no parental data, even though some of our families had more than two siblings, and a few families had parental data. Genetic effects are captured by the additive genetic variance (we assumed no dominance variance), which we varied, and the sibling correlation, which we estimated to be 0.29 for transformed CGN scores in our data. Because of scaling of variances, the additive genetic variance can be interpreted as heritability at the locus (or the proportion of trait variance attributable to the locus). Our calculations used a minor allele frequency of 0.5.

**Linkage:** We computed power to detect suggestive linkage (LOD ≥ 2.2, equivalent to *p* < .0007) and genome-wide significant linkage (LOD ≥ 3.6, equivalent to *p* < 2.3 × 10^-5^), with additive variance ranging from 0.05 to 0.5 (Supplementary Figure 7). We found that we had moderate power for larger values of additive genetic variance. We note that for linkage tests, unlike association tests, this is the additive genetic variance of all the alleles at the locus. These calculations reflect power to detect linkage to a single, specific genetic locus. In the presence of multiple linked regions, the power to detect one or more of the loci goes up considerably [(1 - (1 - β) ^*k*), where *k* is the number of independent loci, and β is the power of a single locus]. Additionally, this power is an underestimate, since we simplified to sibling pairs, ignoring information inherent in the parental information and a limited number of larger sibships.

**Family-based association:** We estimated the power to detect association at genome-wide significant (*p* < 5 × 10^-8^) and suggestive (*p* < 1 × 10^-5^) levels of association. We found that we had good power to detect association for a locus with additive variance at least 0.10, i.e., explaining 10% of the variance (Supplementary Figure 8). Heritability of GWAS loci for many complex traits is typically very small (e.g., for Alzheimer’s disease the well-established risk factor APOE has a genetic variance of ~0.06 and other known SNPs have genetic variance ~0.02 (Ridge, Mukherjee, Crane, Kauwe, & Alzheimer's Disease Genetics, 2013)), so an additive variance more on the order of 0.025 might be more realistic, having power only of about 0.13 for suggestive association. However, we also might assume that for a complex trait, such as CGN, polygenicity is the norm; therefore, we would expect several such loci across the genome. The power to detect association at any one of these loci significantly increases. Taken together, this means that we have likely found some true associations, but also likely have missed some. This highlights the need for replication and new discoveries in larger datasets.
